# Requirement for PRC1 subunit BMI1 in host gene activation by Epstein–Barr virus protein EBNA3C

**DOI:** 10.1093/nar/gky1323

**Published:** 2019-01-15

**Authors:** Kostas Paschos, Quentin Bazot, Jonathan Lees, Paul J Farrell, Martin J Allday

**Affiliations:** 1 Molecular Virology, Department of Medicine, Imperial College London, London W2 1PG, UK; 2 Oxford Brookes University, Faculty of Health and Life Sciences, Oxford OX3 0BP, Oxfordshire, UK

## Abstract

Epstein–Barr virus proteins EBNA3A, EBNA3B and EBNA3C control hundreds of host genes after infection. Changes in epigenetic marks around EBNA3-regulated genes suggest that they exert transcriptional control in collaboration with epigenetic factors. The roles of polycomb repressive complex (PRC)2 subunit SUZ12 and of PRC1 subunit BMI1 were assessed for their importance in EBNA3-mediated repression and activation. ChIP-seq experiments for SUZ12 and BMI1 were performed to determine their global localization on chromatin and analysis offered further insight into polycomb protein distribution in differentiated cells. Their localization was compared to that of each EBNA3 to resolve longstanding questions about the EBNA3–polycomb relationship. SUZ12 did not co-localize with any EBNA3, whereas EBNA3C co-localized significantly and co-immunoprecipitated with BMI1. In cells expressing a conditional EBNA3C, BMI1 was sequestered to EBNA3C-binding sites after EBNA3C activation. When SUZ12 or BMI1 was knocked down in the same cells, SUZ12 did not contribute to EBNA3C-mediated regulation. Surprisingly, after BMI1 knockdown, EBNA3C repressed equally efficiently but host gene activation by EBNA3C was impaired. This overturns previous assumptions about BMI1/PRC1 functions during EBNA3C-mediated regulation, for the first time identifies directly a host factor involved in EBNA3-mediated activation and provides a new insight into how PRC1 can be involved in gene activation.

## INTRODUCTION

Epstein–Barr virus (EBV) is a herpesvirus that asymptomatically infects most of the human population. It is also the causative agent of benign lymphoproliferative disease infectious mononucleosis (1) and is associated with several malignancies, mainly of B-cell origin, but also epithelial nasopharyngeal carcinomas and gastric carcinomas (2–4). EBV has a strong tropism for resting B cells and its life cycle is tightly associated with B-cell differentiation.

According to the current model of EBV persistence *in vivo* (5,6), newly infected resting B cells are induced to proliferate by the growth program of the virus, a transcriptional program during which nine viral proteins are expressed (six nuclear antigens—EBNAs 1, 2, 3A, 3B, 3C and LP—and three membrane proteins—LMP1, LMP2A and LMP2B) together with several RNA species. Activation of B cells is a necessary step in the life cycle of the virus, imitating normal B-cell differentiation, which probably requires passage of the infected cells through the germinal centre on their way to becoming latently infected, resting memory B cells, where viral gene expression is shut down. All nuclear antigens act to affect transcription in a way that allows or facilitates B-cell activation and, by extension, the viral life cycle. EBNA3A, EBNA3B and EBNA3C studied here have been shown to affect the expression of thousands of host genes, with EBNA3A and EBNA3C together able to act as repressors or activators and EBNA3B seemingly acting only as repressor (7).

The EBNA3s control genes epigenetically. Changes in histone acetylation have been observed at the promoters of all EBNA3-control genes tested, with repressed genes losing acetylation and activated genes gaining acetylation concurrently with changes in expression (reviewed in (7)). Numerous studies have also described EBNA3-repressed genes that are characterized by the presence of H3K27me3, the repressive epigenetic mark deposited by polycomb repressive complex (PRC)2 (8–15). PRC2 and the H3K27me3 mark can act as precursors for DNA methylation (16), which in turn can repress tumour suppressor genes in cancer (17–19). It has already been suggested that for EBNA3-repressed tumour suppressor gene BCL2L11 (expressing the pro-apoptotic member of BCL-2 family, BIM) H3K27me3 deposition in the presence of EBNA3A and EBNA3C could lead to DNA methylation (12,20) that makes infected cells resistant to aberrant expression of Myelocytomatosis proto-oncogene (MYC) and provides an obvious path to EBV-associated endemic Burkitt’s lymphoma (reviewed in (4)). Due to the potential importance of PRC2 in EBNA3-mediated regulation and the potential consequences of PRC2 involvement, we sought to address important unanswered questions about the role of this protein complex. ChIP-seq studies for the discovery of genomic binding of the EBNA3s indicate that H3K27me3 is not found at EBNA3 binding sites (21,22), but the same studies showed that EBNA3s bind to regions distal to the genes they regulate and therefore it is possible that PRC2 is found closer to the promoters of EBNA3-regulated genes, rather than the EBNA3-binding sites. H3K27me3 deposition, as judged by ChIP-QPCR for specific loci around EBNA3C-repressed genes, comes after the first occurrence of repression for a few genes tested (10,15). It is unclear if H3K27me3 occurs at other loci concurrently with repression or whether low levels of H3K27me3 detected at these loci at the beginning of repression still contribute to repression establishment. For the first time, we assessed the presence of PRC2 at EBNA3-binding sites and at the promoters they regulate. SUZ12 was used as a proxy for PRC2, because it is a core subunit that is essential for complex assembly on to chromatin, deposition of H3K27me3 (23), and was previously found at EBNA3-regulated loci (8–10,12). We also tested how repression establishment is affected after impairment of PRC2 by SUZ12 knockdown and PRC2’s contribution to repression maintenance after 21 days of EBNA3 action. Taken together, experiments presented here clarify the role of PRC2 at EBNA3-regulated genes and the extent of its influence genome-wide.

PRC1 was originally thought to be recruited to chromatin by PRC2, because polycomb (Pc) subunits in *Drosophila* (and human homologues CBX) were found to bind strongly to H3K27me3 (24,25). For this reason, experiments that indicated a relationship between EBNA3s and PRC2, also put a focus on PRC1 relative to the EBNA3s as an important complex for repression, differentiation and specifically haematopoiesis (26–28). The catalytic subunits of PRC1, RING1B and RING1A (also known as RING2 and RING1 in human) exhibit E3 ubiquitin ligase activity and implement mono-ubiquitination on H2AK119, an epigenetic mark that is important for the repressive action of the complex (29).

Canonical PRC1 complexes contain members of the CBX family of proteins (CBX1–8) that show strong affinity to H3K27me3 or H3K9me3 due to a conserved C-terminal chromodomain (30–32). CBX proteins bind to RING1A or RING1B via an N-terminal PcG box (33–35), whereas RING1A/B also binds to polycomb group RING finger proteins (PCGF1–6) that enhance the E3 ligase activity of the catalytic subunits (36–38). Previous experiments indicated that of the PCGF proteins, PCGF4, also known as BMI1, might be important in EBNA3-mediated repression, because BMI1 knockdown in a lymphoblastoid cell line (LCL) resulted in some de-repression of EBNA3A- and EBNA3C-repressed *BIM* (12). Supporting this hypothesis, BMI1 has also been found to accumulate with EBNA3C around EBNA3C-repressed promoters (10). In this study, we assessed the role of PRC1’s BMI1 in EBNA3-mediated regulation, first by investigating the global distribution of BMI1 on chromatin and relating this to the localization of each EBNA3 and then by testing the effects of BMI1 knockdown on EBNA3 regulation. Our results overturn previous assumptions about the importance of BMI1 on EBNA3-mediated repression and reveal a surprising and consistent role in activation, making BMI1 the first factor directly shown to be needed for EBNA3-mediated activation and providing the first paradigm for host gene activation by EBV with polycomb complex involvement.

## MATERIALS AND METHODS

### Cell culture

LCLs were grown at 37°C in 10% CO_2_ with Roswell Park Memorial Institute (RPMI)-1640 medium (Invitrogen), supplemented with 10% foetal bovine serum penicillin and streptomycin. Puromycin was used at 1 μg/ml to select cells stably transduced with lentiviruses. 4-Hydroxytamoxifen (HT) was used at 400 nM and doxycycline at 500 ng/ml, where indicated. All supplements were added only to the fresh media added to cultures as needed. Adherent 293T cells were cultured in DMEM (Invitrogen), supplemented with 10% foetal bovine serum, penicillin and streptomycin.

### Western blots, co-immunoprecipitations and RT-QPCR

Western blots and immunoprecipitations were performed as described previously (12). Antibodies for western blot were anti-EBNA3A (Abcam, ab16126, 1:1000 dilution), anti-EBNA3B (clone 6C9, Allday lab, E. Kremmer (39), 1:10 dilution), anti-EBNA3C (clone A10, gift from M. Rowe, University of Birmingham, 1:10 dilution), anti-γ-tubulin (Sigma, T6557, 1:8000 dilution), anti-BMI1 (Millipore, 05–637, 1:1000 dilution), anti-SUZ12 (Santa Cruz Biotechnology, sc-46264, 1:1000 dilution), anti-CBX4 (Santa Cruz, sc-517216, 1:1000 dilution) and anti-MEL18 (Abcam, ab5267, 1:1000 dilution). Antibodies for immunoprecipitations were anti-BMI1 (Bethyl Laboratories, A301-694A, 2 μg) and DYKDDDDK Tag antibody (NEB, 2368, 2 μg). RNA extraction was performed using Qiagen’s RNeasy mini kit, according to the manufacturer’s instructions. cDNA was obtained using Invitrogen’s SuperScript III First Strand Synthesis Supermix. QPCR for cDNA and DNA from ChIP was performed using Platinum SYBR green QPCR Supermix uracil DNA glycosylase (UDG) kit (Invitrogen), as described previously (12). Primers for STK39 (8), AICDA (40) and COBLL1 (10) loci and expression have been described before. All the oligonucleotide primers used are listed in Supplementary Table S1.

### Chromatin immunoprecipitations

ChIP was performed as described previously (22). Briefly, 15 × 10^6^ cells were fixed for 10 min in 1% formaldehyde at room temperature and then washed, resuspended in swelling buffer (25 M 4-(2-hydroxyethyl)-1-piperazineethanesulfonic acid (HEPES), pH 7.8; 1.5 mM MgCl_2_; 10 mM KCl, 0.1% 4-Nonylphenyl poly(ethylene glycol) (NP-40); 1 mM DL-Dithiothreitol (DTT); 1 mM phenylmethylsulfonyl fluoride (PMSF); 1 μg/ml aprotinin; 1 μg/ml pepstatin A) and incubated at 4°C for 20 min with rotation. The swelling buffer was aspirated after centrifugation. The pelleted nuclei were resuspended in 1 ml sonication buffer (50 mM HEPES, pH 7.9; 140 mM NaCl; 1 mM ethylenediaminetetraacetic acid (EDTA); 1% Triton X-100; 0.1% sodium deoxycholate; 0.1% sodium dodecyl sulphate; 1 mM PMSF; 1 μg/ml aprotinin; 1 μg/ml pepstatin A), incubated on ice for 30 min and then sonicated for 1 h with a Covaris M220 Focused-ultrasonicator with milliTUBE holder (peak power 75; duty factor 26; cycles/burst 200; temperature 6°C). The lysate was centrifuged at 10000g for 10 min at 4°C and the 1 ml of supernatant was diluted with 3.2 ml of sonication buffer. Two hundred microlitres of the sonicated lysate were taken for input control. Antibody/magnetic bead incubations and washing steps were performed as described previously (22) in 15 ml Falcon tubes. Antibodies for ChIP were anti-BMI1 (Bethyl Laboratories, A301-694A, 16 μg), anti-SUZ12 (Abcam, ab12073, 16 μg) and Normal Rabbit IgG (Millipore, PP64B, 16 μg).

### Next-generation sequencing

DNA from four independent chromatin immunoprecipitations was pooled for each factor and was run on a 2% agarose gel, as described previously (22), to isolate fragments between 100 and 500 bp using Qiagen’s MinElute gel purification kit. More than 5 ng of DNA for each sample was sent to the Harvard Biopolymers facility for sequencing after library construction (Illumina HiSeq 2500, 50 bp single reads). The same was done from the relevant input controls, which were non-precipitated chromatin from all chromatin immunoprecipitations pooled together.

### ChIP-seq data analysis

Sequenced reads were aligned to the human genome version hg19 using BWA (41). A total of 33.9 × 10^6^ uniquely mapped reads were obtained from the input sample, 29 × 10^6^ from the SUZ12 sample and 27.2 × 10^6^ from the BMI1 sample. Peaks for each factor were called using the MACS algorithm (42). Peaks with a *q* value cut-off of 5.00e-02 were used for downstream analyses and are listed in Supplementary File S1. Partek® software, Version 6.6 was used to determine peak co-localization with peaks deemed co-localized if they had 1 or more common bp. Publicly available ChIP-seq tracks used in this study are listed in Supplementary File S2. The EBNA3-regulated genes considered are the same as used previously (22). Promoters of genes were defined as the region 1000 bp upstream to 500 bp downstream of each transcription start site (TSS). Pearson’s chi-squared test was performed using a 2 × 2 contingency table (43).

### Production of shRNA-expressing lentiviruses and lentiviral transduction

ShRNAs were based on sequences from The RNAi Consortium (TRC, https://www.broadinstitute.org/rnai-consortium). Oligonucleotides were annealed for the stem sequence of each shRNA and cloned into pLKO.1 (Addgene plasmid 10878; (44)) or Tet-pLKO-PURO (Addgene plasmid 21915; (45)) to create plasmids for lentivirus-based expression in LCL. The oligonucleotides used were: non-targeting (from TRC SHC002V) forward: 5′-CCGGCCTAAGGTTAAGTCGCCCTCGCTCGAGCGAGGGCGACTTAACCTTAGGTTTTTG-3′, reverse: 5′-AATTCAAAAACCTAAGGTTAAGTCGCCCTCGCTCGAGCGAGGGCGACTTAACCTTAGG-3′; BMI1 (from TRCN0000020155) forward: 5′-CCGGCCAGACCACTACTGAATATAACTCGAGTTATATTCAGTAGTGGTCTGGTTTTTG-3′, reverse: 5′-AATTCAAAAACCAGACCACTACTGAATATAACTCGAGTTATATTCAGTAGTGGTCTGG-3′; SUZ12 (from TRCN0000038728) forward: 5′-CCGGGCTTACGTTTACTGGTTTCTTCTCGAGAAGAAACCAGTAAACGTAAGCTTTTTG-3′, reverse: 5′-AATTCAAAAAGCTTACGTTTACTGGTTTCTTCTCGAGAAGAAACCAGTAAACGTAAGC-3′. Ten micrograms of each of these plasmids were transfected together with 8 μg of psPAX2 (Addgene plasmid 12260; a gift from Didier Trono) and 2 μg of pMD2.G (Addgene plasmid 12259; a gift from Didier Trono). Transfections were performed by the calcium phosphate precipitate method. Briefly, ∼2.5 × 10^6^ 293T cells were seeded in a 10 cm cell culture dish. On the following day, the amounts of plasmids given above were mixed and H_2_O was added to 450 μl. Fifty microlitres of 2 M CaCl_2_ was added to the DNA solution and the new mix was added to 500 μl of 2× HEPES buffered saline (2× HBS, pH 7.1) and left at room temperature for 30 min. The DNA/CaCl_2_/HBS solution was taken up without mixing and added dropwise to the plates with the 293T cultures. After 8 h incubation the medium was aspirated, the cells washed with sterile 1× PBS and 5 ml of fresh medium were added. The lentivirus-containing medium was harvested 2 days later, passed through a 45 μm filter and stored at −80°C.

For lentiviral transduction, LCL cells were incubated with 8 μg/ml polybrene for 15 min, then 10 × 10^6^ cells were pelleted by centrifugation, resuspended in 500 μl of lentivirus-containing medium and centrifuged for 1.5 h at 450g at 20°C. The lentivirus-containing medium was then aspirated and the cells resuspended in 3 ml of RPMI medium and transferred in flasks. Puromycin selection was added 48 h after lentiviral infection to obtain stably transduced cell lines.

## RESULTS

### BMI1 associates with regulatory elements, whereas SUZ12 associates with regions characteristic of polycomb regulation

**Figure 1.**
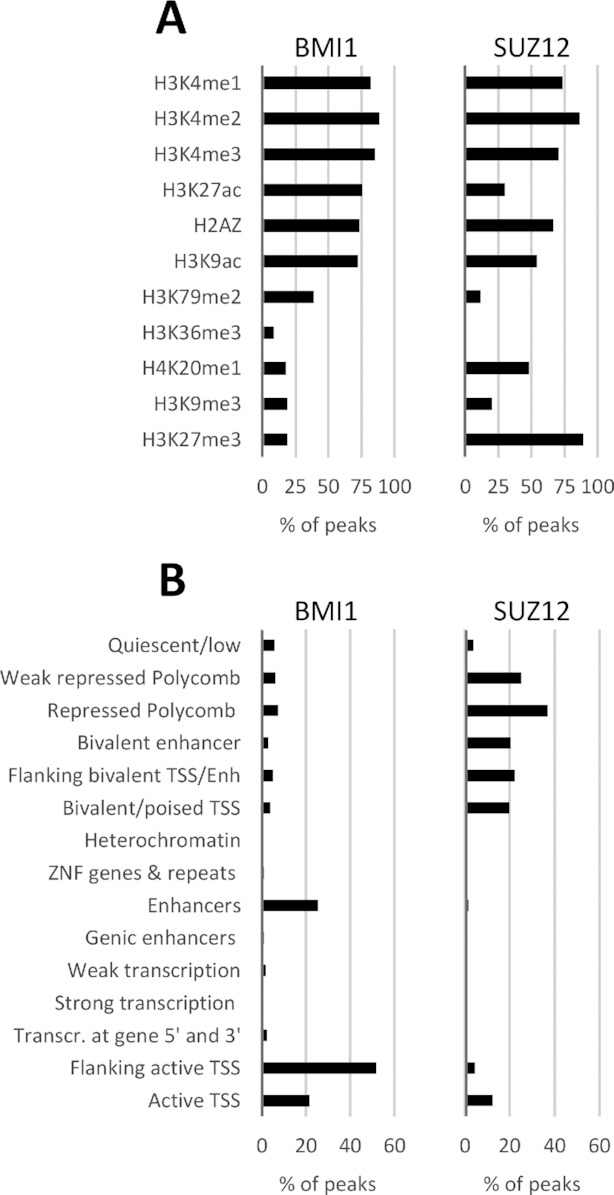
Association of BMI1 and SUZ12 peaks with histone modifications and chromatin states. (**A**) Co-localization of BMI1 and SUZ12 peaks, as determined by the MACS algorithm from ChIP-Seq performed for this study, with histone modifications in LCL GM12878 determined by the ENCODE project. The length of each histogram bar represents the percentage of peaks co-localizing with regions containing each histone modification. Peaks were considered co-localized if they had 1 or more bp in common. Many peaks co-localize with more than one histone modification mark, because at many genomic regions more than one modification is present. (**B**) Co-localization of BMI1 and SUZ12 peaks with genome segments corresponding to 15 chromatin states characterized by the Roadmap Epigenomics consortium.

ChIP-seq was performed to study the localization of BMI1 and SUZ12 across the host genome in an LCL produced by infection of 1^o^ B cells with a recombinant EBV of B95.8 background (prototypical transforming EBV, originally derived from an infectious mononucleosis patient). The 1^o^ B cells came from the same donor as that used in a previous study (22) to identify genomic localization of EBV latent proteins EBNA3A, EBNA3B and EBNA3C. 7623 BMI1 and 1589 SUZ12 peaks were identified using the MACS algorithm (Supplementary File S1). Extensive ChIP-seq data are available for an LCL (GM12878) from the ENCODE project [https://www.encodeproject.org, (46)], which include uniformly processed experiments with peaks called for 11 histone modifications. These were used to determine the association of BMI1 and SUZ12 with regions that also contain each of these modifications (Figure 1A). BMI1 was found to associate mostly with histone modifications characteristic of active enhancers (H3K27ac and H3K4me1) or commonly found at promoters of actively transcribed genes (H3K4me2, H3K4me3, H3K27ac, H3K9ac and histone variant H2A.Z) (47). These BMI1–histone modification associations were reminiscent of those seen for EBNA3A and EBNA3C previously (22). It is not surprising that the sum of all BMI1 co-localization percentages is greater than 100 because many histone modifications are found at the same regions of the genome. SUZ12 co-localized with some of the same modifications as BMI1, such as H3K4me1/2/3 and H2A.Z. Importantly, the highest co-localization (over 90%) was with H3K27me3 (Figure 1A), the epigenetic mark deposited by the SUZ12-containing PRC2, clearly indicating the validity of the ChIP-seq experiment. In a marked difference, SUZ12 is associated more than BMI1 with H4K20me1 and less than BMI1 with H3K27ac, characteristic of active enhancers and promoters (Figure 1A).

The histone modifications discussed above, together with global DNA methylation and DNA accessibility data, were used by the Roadmap Epigenomics consortium (48) (Supplementary File S2) to assign regions of the LCL GM12878 genome to 1 of 15 chromatin states. Confirming the trends seen just with the histone modification data, BMI1 peaks were found co-localized mostly with regions characterized as enhancers, active TSS or flanking active TSS (Figure 1B). SUZ12 localized mostly to regions characterized as weakly repressed by polycomb, repressed by polycomb and bivalent regions (enhancers and TSS) (Figure 1B), all expected for a subunit of PRC2 (49), but different to trends seen previously for the EBNA3 proteins.

### BMI1 co-localizes better with EBNA3C than with EBNA3A or EBNA3B; SUZ12 does not co-localize with the EBNA3 proteins

**Figure 2.**
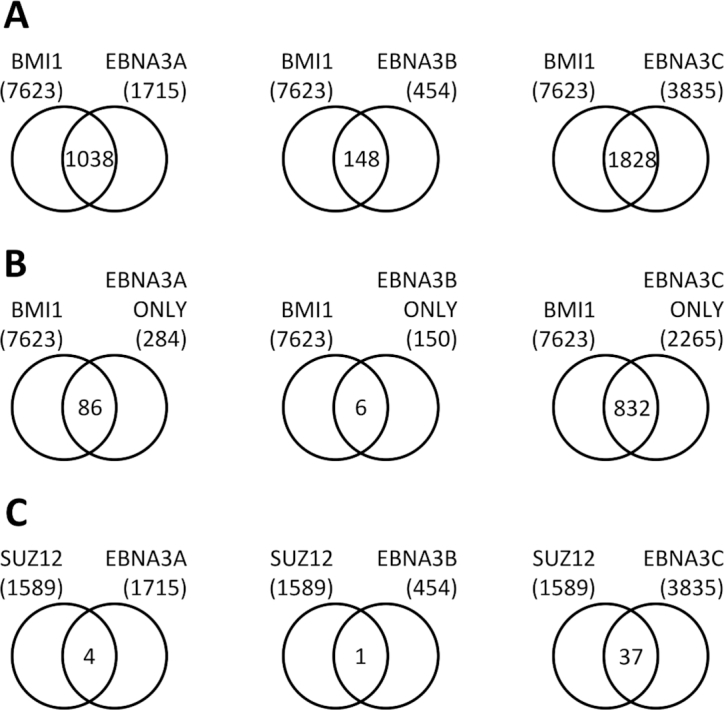
Co-localization of BMI1 and SUZ12 peaks with EBNA3A, EBNA3B and EBNA3C peaks. (**A**) Co-localization of BMI1 peaks determined in this study with EBNA3A, EBNA3B or EBNA3C peaks determined in a previous study (22). Peaks were considered co-localized if they had one or more bp in common. (**B**) Co-localization of BMI1 peaks and regions with only one EBNA3 (EBNA3A-, EBNA3B- or EBNA3C-only peaks). (**C**) Co-localization of SUZ12 peaks determined in this study with EBNA3A, EBNA3B or EBNA3C peaks.

Co-localization between BMI1 or SUZ12 peaks and EBNA3A, -3B or -3C peaks [(22) and Supplementary File S1] was assessed. When considering the total number of peaks for EBNA3A, EBNA3B and EBNA3C, there is considerable co-localization of all with BMI1 (Figure 2A). The most co-localized with 1038 out of 1715 peaks (∼61%) is EBNA3A, followed by EBNA3C with 1828 out of 3835 peaks co-localized (∼48%) and then EBNA3B with 148 out of 454 (∼33%).

However, it has been shown previously that the EBNA3s can interact with each other (12) and that they significantly co-localize with each other. Therefore, when looking at total numbers of peaks for each EBNA3, many sites considered are occupied by more than one EBNA3 and it is possible that co-localization with BMI1 is not due to all EBNA3s present at each locus. To examine the level of co-localization of each EBNA3 with BMI1, without the functional interference of the other EBNA3s, the localization of EBNA3A-only, EBNA3B-only or EBNA3C-only peaks with BMI1 peaks was examined. These are loci where only one EBNA3 is present without evidence of presence for the other two (22). In this comparison (Figure 2B), it is EBNA3C that appears to be most closely associated with BMI1, with 832 out of 2265 EBNA3C-only peaks co-localized with BMI1 peaks (∼37%). EBNA3B does not significantly co-localize, with only 6 out of 150 EBNA3B-only peaks co-localizing with BMI1 (4%). About 30% of EBNA3A-only peaks co-localize with BMI1 peaks (86 out of 284).

There is minimal SUZ12 peak co-localization with EBNA3 peaks, the highest being 37 out of a total 1589 (2.3%) SUZ12 peaks co-localizing with EBNA3C, suggesting that there is no direct correlation between regions of SUZ12 and EBNA3 binding (Figure 2C). However, it is known that EBNA3 proteins bind mostly to sites distal to the promoters they regulate (21,22) and therefore it is possible that SUZ12 binds to promoters of EBNA3-regulated genes, rather than EBNA3-binding sites.

### BMI1 peaks are found more frequently at loci of EBNA3A and EBNA3C peaks, rather than EBNA3A- or EBNA3C-regulated promoters

The co-localization of BMI1 and SUZ12 peaks with EBNA3 peaks or with promoters of host genes EBNA3s regulate was compared. To do this, BMI1 or SUZ12 peaks within contact domains that contained both EBNA3-regulated genes and EBNA3 peaks were considered. Contact domains are regions in the LCL genome that were found to come into contact via looping with high frequency (50). It is assumed that EBNA3 peaks regulate directly EBNA3-regulated genes found on the same contact domain because they can come into direct contact via chromatin looping. Contact domains containing up-regulated or down-regulated genes were considered separately, for EBNA3A and EBNA3C. Analysis for EBNA3B is not presented further, because no significant association of EBNA3B was found with either BMI1 or SUZ12.

**Figure 3.**
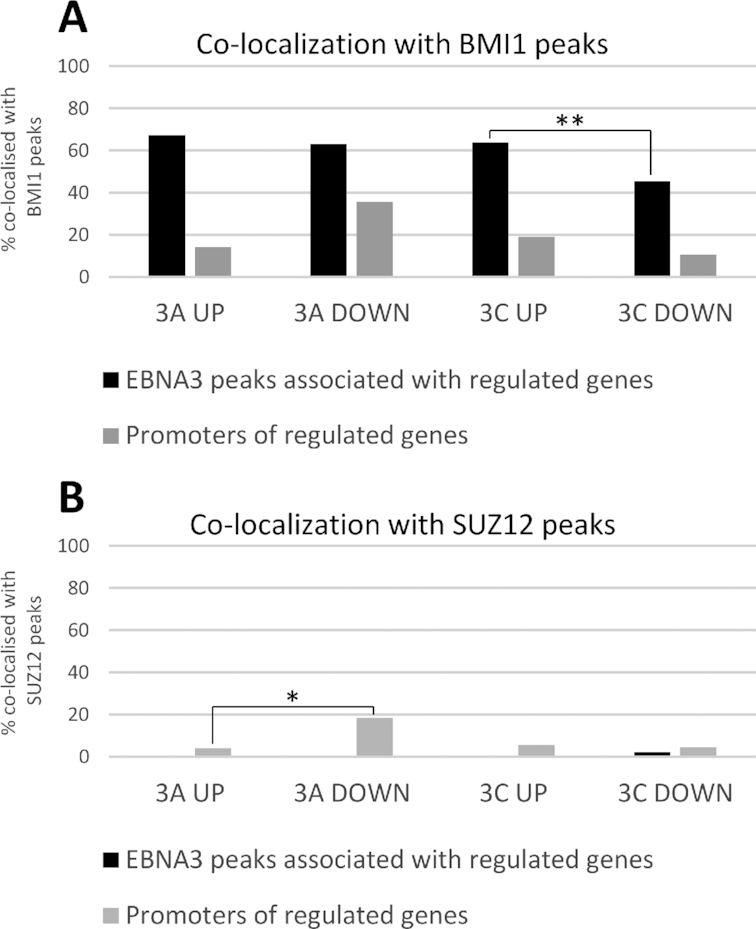
Co-localization of BMI1 and SUZ12 peaks with promoters of EBNA3-regulated genes and EBNA3 peaks directly associated with EBNA3-regulated genes. (**A**) Percentage of BMI1 peaks co-localized with EBNA3A or EBNA3C peaks directly associated with EBNA3A/C up- or down-regulated genes (black bars) and BMI1 peaks co-localized with promoters of EBNA3A/C-up- or down-regulated genes (grey bars). EBNA3A/C directly regulated genes determined previously (22). Promoters defined as the region from 500 bp downstream to 1000 bp upstream of the transcription start site of each gene. Peaks were considered as directly associated with EBNA3-regulated genes if found within contact domains that also contained the EBNA3-regulated gene’s transcription start site. Pearson’s chi-squared test indicated statistically significant difference (*P* < 0.01) where indicated (**). (**B**) Same co-localization analysis as in (A), but for SUZ12 peaks. Pearson’s chi-squared test indicated statistically significant difference (*P* < 0.05) where indicated (*).

BMI1 was found to co-localize more frequently with EBNA3A and EBNA3C peaks, rather than with the promoters of genes these EBNA3s regulate (Figure 3A). This was the case for EBNA3A and EBNA3C peaks. Unexpectedly, BMI1 co-localized significantly (*P* < 0.01) more with EBNA3C peaks in contact domains containing EBNA3C-activated genes than containing EBNA3C-repressed genes (Figure 3A).

SUZ12 was again shown to not co-localize with EBNA3 peaks, as previously, but there was also very little co-localization with EBNA3-regulated promoters. The highest incidence of co-localization was for EBNA3A-down-regulated promoters at slightly lower than 20% (Figure 3B), which is significantly (*P* < 0.05) higher than co-localization for EBNA3A-up-regulated promoters.

### Only EBNA3C can co-immunoprecipitate significantly with BMI1

**Figure 4.**
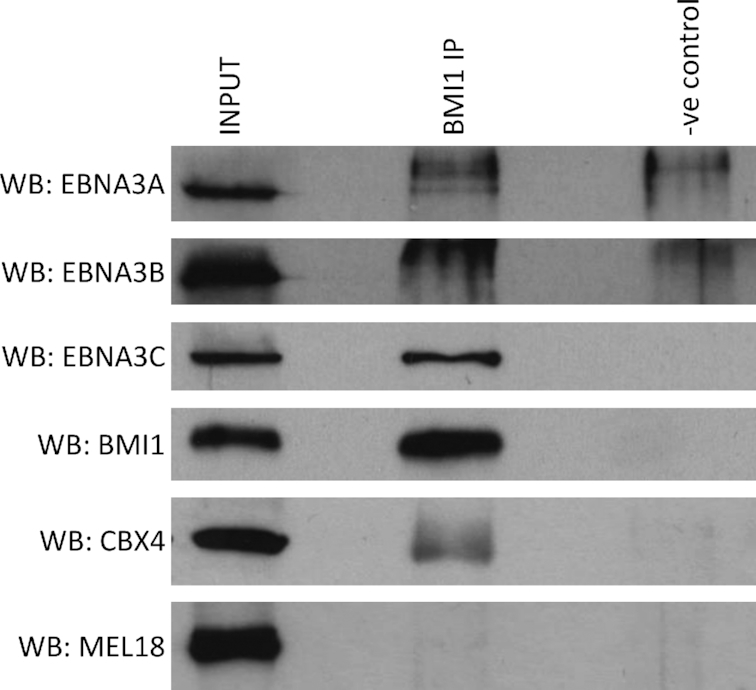
EBNA3C can be co-immunoprecipitated with BMI1 in LCL. A rabbit anti-BMI1 antibody and—as a negative control—a rabbit anti-FLAG antibody were used for immunoprecipitations. The precipitates were run on sodium dodecylsulphate-polyacrylamide gel electrophoresis gels, western blotted and probed for EBNA3A, EBNA3B, EBNA3C; for BMI1 or CBX4 as a positive control and for MEL18 as a negative control. About 5% of input sample is shown for comparison. A representative example of more than three independent experiments is shown.

Computational analysis of ChIP-seq data for BMI1, SUZ12 and the EBNA3s suggested a close relationship between BMI1 and EBNA3C and possibly between BMI1 and EBNA3A on chromatin. To test which of the EBNA3s could be found in complexes with BMI1, an anti-BMI1 antibody was used to immunoprecipitate BMI1 from extracts from LCLs infected with prototypical B95.8 EBV (Figure 4). EBNA3C was found to co-immunoprecipitate reliably with BMI1, whereas very little EBNA3A and no EBNA3B co-immuprecipitated (Figure 4).

Similar co-immunoprecipitation experiments using two different anti-SUZ12 antibodies failed to show a physical interaction with any of the EBNA3s, with none of them co-immunoprecipitated (data not shown).

### EBNA3C increases BMI1 occupancy at EBNA3C peaks

Since BMI1 was found at EBNA3A and EBNA3C peak loci and at least EBNA3C was able to be in complex with BMI1, the effect of EBNA3A and EBNA3C on BMI1 recruitment onto chromatin was tested. Two LCLs expressing either a conditional EBNA3A or a conditional EBNA3C were used. The LCL expressing conditional EBNA3C is *p16^INK4A^*-null, because in p16^INK4A^-competent LCLs inactivation of EBNA3C leads to *p16^INK4A^* induction and cell arrest, whereas *p16^INK4A^*-null cells can grow without EBNA3C (51). The conditional EBNA3C expressed (3CHT) is fused to a modified oestrogen receptor that renders 3CHT active only in the presence of HT (13). In the absence of HT, 3CHT is sequestered to the cytoplasm and degraded. HT had never been added to the medium since 1^o^ B cells were infected with recombinant viruses, therefore active EBNA3C was never present in these cells before the experiment to be described. The conditional EBNA3A LCL expresses EBNA3A fused to a newer version of the modified oestrogen receptor, termed ERT2 (3AERT2) (52,53). This LCL was also grown out without the addition of HT in the medium, which is possible because EBNA3A-null EBV can transform B cells, albeit with reduced efficiency (54).

**Figure 5.**
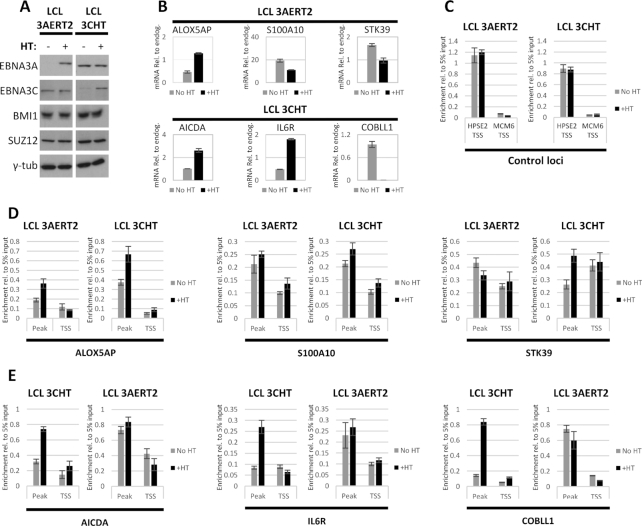
BMI1 is recruited to EBNA3-regulated genes by EBNA3C. LCL infected with recombinant EBV expressing either a conditional EBNA3A (3AERT2) or a conditional EBNA3C (3CHT) activated by adding HT to the culture medium. For both LCLs, HT had never been added to the medium prior to experiment shown. HT was added to half the culture of each LCL and cells grown with or without HT were harvested 14 days after HT addition. Representative results from one of three independent experiments are shown. (**A**) Activation and stabilization of 3AERT2 and 3CHT were verified by western blotting. BMI1 and SUZ12 protein levels did not change after HT addition. Γ-Tubulin was used as loading control. (**B**) RT-QPCR was performed to assess mRNA amounts and confirm that EBNA3A-regulated genes were activated (ALOX5AP) or repressed (S100A10 and STK39) as expected after addition of HT in the 3AERT2 LCL and EBNA3C-regulated genes were activated (AICDA and IL6R) or repressed (COBLL1) as expected after HT addition in the 3CHT LCL. Height of bars indicates mRNA levels, normalized to mRNA levels of endogenous control gene, GNB2L1. Error bars represent standard deviation from three QPCR replicates. (**C**) ChIP for BMI1 was carried out for No HT and +HT 3AERT2 and 3CHT cultures. Enrichment at a locus identified to be positive for BMI1 from the ChIP-seq experiment, but without evidence of significant EBNA3 occupancy was used as a positive control. No evidence of BMI1 or EBNA3 binding was found at MCM6 TSS from ChIP-Seq experiments and this site was used as negative control for the BMI1 ChIP. Height of bars represents enrichment relative to 5% of input chromatin. Error bars show standard deviation from three QPCR replicate reactions. (**D**) ChIP as in (C) showing enrichment of BMI1 at genes found to be up-regulated (ALOX5AP) or down-regulated (S100A10, STK39) by EBNA3A. Occupancy was assessed at the TSS of each gene and the locus directly associated with the TSS found to have the highest BMI1 peak by ChIP-Seq, for LCL 3AERT2 and LCL 3CHT. (**E**) Same as (D) for genes found to be up-regulated (AICDA, IL6R) or down-regulated (COBLL1) by EBNA3C.

HT was added to half the culture of LCL 3AERT2 and LCL 3CHT HT and all cultures were grown in parallel for 14 days, at which point cells were harvested. This time point was chosen because it was shown previously in a similar experiment with LCL 3CHT that BMI1 accumulated around that time at two EBNA3C-repressed loci (10). The stabilization of 3AERT2 and 3CHT in response to HT addition was verified by western blot, as was the fact that HT addition did not affect BMI1 or SUZ12 protein levels (Figure 5A). Three EBNA3A-regulated gene loci (one up- and two down-regulated) (39,54) (Supplementary Figure S1A) and three EBNA3C-regulated gene loci (two up- and one down-regulated) (39,51) (Supplementary Figure S1B) were selected because from the ChIP-seq experiments they were all found to have significant BMI1 and EBNA3 peaks. mRNA for all these genes was assessed by quantitative reverse transcription PCR (RT-QPCR) and they were all found to be regulated by 3AERT2 and 3CHT as expected by 14 days post-addition of HT (Figure 5B). The endogenous control gene GNB2L1, used to normalize values, was not affected by the addition of HT during the time course for either LCL (Supplementary Figure S2A).

Loci with no significant EBNA3A or EBNA3C binding (TSS of HPSE2, positive for BMI1 binding, and TSS of MCM6, negative for BMI1 binding) (Supplementary Figure S2B) were used as controls for BMI1 ChIP-QPCR. Addition of HT had no effect on BMI1 occupancy at these loci during the 14-day time course for either LCL 3AERT2 or LCL 3CHT (Figure 5C).

BMI1 enrichment was compared between cells cultured in the absence and in the presence of HT, around the selected EBNA3A-regulated genes for both LCL 3AERT2 and LCL 3CHT (Figure 5D). Two loci were tested for each gene: the TSS and the locus of the highest BMI1 peak within the contact domain that contained the regulated gene (Supplementary Figure S1A). For genes S100A10 and STK39, there was no significant recruitment of BMI1 in response to 3AERT2 activation in the LCL 3AERT2, but there was some enrichment in response to 3CHT activation in LCL 3CHT (Figure 5D). At the ALOX5AP locus, there was enrichment of BMI1 in response to both 3AERT2 and 3CHT activation (Figure 5D). It should be noted that at all these EBNA3A-regulated loci EBNA3C is present (Supplementary Figure S1A) and that ALOX5AP, where there is BMI1 recruitment by 3AERT2, is also robustly regulated by 3CHT (Supplementary Figure S2C).

When BMI1 enrichment was compared in a similar manner around the selected EBNA3C-regulated genes, in all cases occupancy increased robustly at the BMI1 peak in response to 3CHT activation (Figure 5E). No significant increase in BMI1 occupancy was observed after 3AERT2 activation at any of those loci (Figure 5E), despite apparent EBNA3A binding at two of the loci [AICDA and COBLL1 (Supplementary Figure S1B)].

### Knockdown of BMI1 leads to activation of EBNA3C-activated genes in the absence of EBNA3C

Following results from peak co-localization, physical interaction and recruitment on to chromatin assessments that suggested EBNA3C has the closest relationship with BMI1, efforts were focused on determining if BMI1 affects regulation by EBNA3C.

From data presented here, SUZ12 does not seem to be closely associated with either EBNA3. However, for EBNA3A, two separate reports point to a more complicated picture. Harth-Hertle *et al.* (9) showed that EBNA3A repression can occur before accumulation of H3K27me3, indicating that this epigenetic mark and thus probably SUZ12, as core component of the complex that implements it, are recruited as a consequence of repression. On the other hand, a recent report (8) showed that for STK39 (the only gene shown to be regulated only by EBNA3A with no EBNA3C functional involvement), PRC2 function is important for repression establishment. It seems that SUZ12 involvement in EBNA3A-mediated regulation (in the very few cases it is present) differs depending on the locus.

We therefore concentrated on the relationships between BMI1 and EBNA3C (in order to develop the findings described so far) and between SUZ12 and EBNA3C, to clarify more thoroughly a relationship that has been suggested previously (12,15,21) but that has also been questioned (10,21).

**Figure 6.**
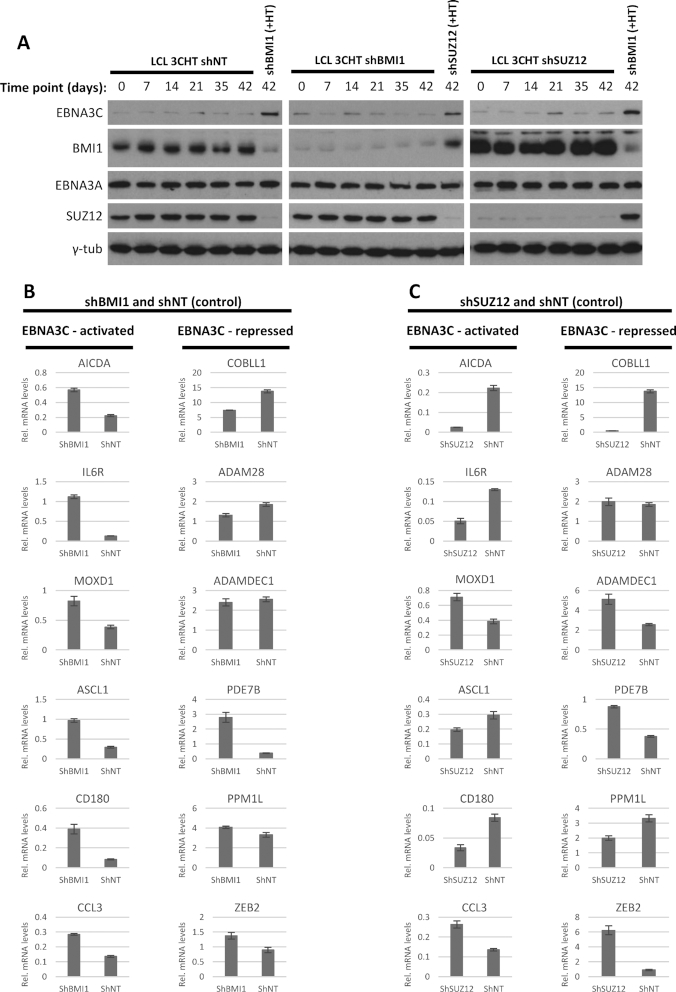
BMI1 and SUZ12 knockdown in LCL not expressing active EBNA3C. BMI1 and SUZ12 were stably knocked down in LCL 3CHT, grown in the absence of HT and thus never having expressed functional HT. Knockdowns were achieved with a lentiviral construct that constitutively expresses shRNA, targeting either BMI1 or SUZ12 (shBMI1 or shSUZ12). A lentivirus expressing a non-targeting shRNA was used as a control (NT). Cells were grown long-term for up to 4 months at a time. (**A**) Western blot confirmed that knockdowns were stable over time. Knockdowns did not affect levels of other proteins tested (EBNA3A, SUZ12 or BMI1 as relevant and loading control γ-tubulin). In each panel, protein from cells grown in the presence of HT and with a different knockdown is shown to help comparison between degraded and stabilized 3CHT and between the different knockdowns. Day 0 is the day HT was added to control cells for the western blot shown. (**B**) Comparison between shBMI1 and shNT. mRNA levels for EBNA3C-activated or -repressed genes as indicated were assessed by RT-QPCR for the 42 days time point. Height of the bars indicates mRNA levels, normalized to mRNA levels for endogenous control GNB2L1. Error bars represent standard deviation from three QPCR replicates. (**C**) As in (B) but for comparison between shSUZ12 and shNT.

To do this, BMI1 or SUZ12 were knocked down stably using lentiviral vectors that constitutively express the relevant shRNAs (shBMI1 and shSUZ12) in LCL 3CHT cells that had always been grown in the absence of HT and therefore in the absence of functional EBNA3C. As a control, a lentivirus stably expressing a non-targeting shRNA (shNT) was also used. The three resultant cell lines were followed, and it was verified by western blotting that both knockdowns were stable over time (Figure 6A). Six genes identified previously (39,51,54) as EBNA3C-activated and six genes identified as EBNA3C-repressed were chosen as examples for study. All genes had associated EBNA3C and BMI1 peaks (Supplementary Figures S1B and S3) and all but one (PDE7B) EBNA3C-repressed genes had associated SUZ12 or H3K27me3 peaks.

QPCR was performed to measure the steady-state levels of mRNA in the absence of HT. The levels were compared between LCL 3CHT shBMI1 and LCL 3CHT shNT. The endogenous control gene GNB2L1 used to normalized values was not affected by the BMI1 knockdown (Supplementary Figure S4A). Surprisingly, it was found that in cells that did not express functional 3CHT all EBNA3C-activated genes were up-regulated following BMI1 knockdown, relative to the non-targeting control (Figure 6B). For EBNA3C-repressed genes, BMI1 knockdown in cells not expressing functional 3CHT had a variable effect with all possible outcomes observed—repression, de-repression and no change, depending on the locus (Figure 6B).

When levels of the same mRNAs were similarly compared between shSUZ12 and shNT cells, for activated or repressed genes again no general trend was observed, with repression or de-repression being observed in a locus-specific manner (Figure 6B). Control gene GNB2L1 expression was not affected by the SUZ12 knockdown (Supplementary Figure S4A).

### In cells with stably knocked down SUZ12, 3CHT can activate and repress efficiently whereas, in cells with stably knocked down BMI1, 3CHT can only repress efficiently

**Figure 7.**
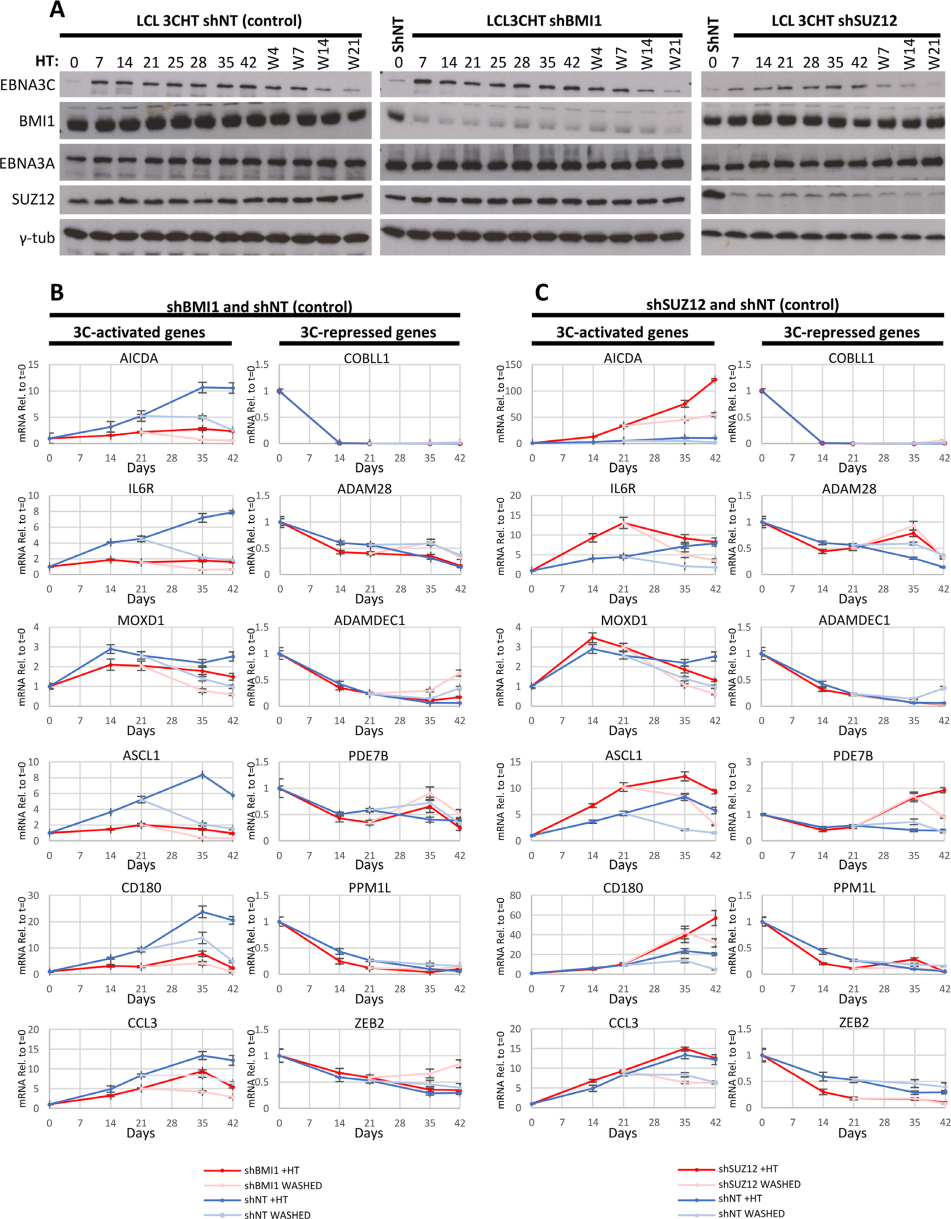
Effect of BMI1 or SUZ12 stable knockdown on host gene regulation by EBNA3C. BMI1 and SUZ12 were stably knocked down in LCL 3CHT, as described in Figure 6. HT was added to half the culture of each of the three cell lines and after 21 days HT was washed from half the cultures that contained HT. All the cultures were grown for up to 42 days after initial addition of HT and samples were taken at time points indicated. Representative results from one of three independent time courses are shown. (**A**) EBNA3C protein activation and stabilization after addition of HT and degradation after washing of HT was confirmed by western blotting. Knockdowns of BMI1 or SUZ12 were also confirmed and verified not to be affected by HT addition. EBNA3A protein levels were also assessed as control and γ-tubulin was used as loading control. Days post-addition of HT are indicated as days post-washing of HT (with ‘W’ prefix). For the blots showing the BMI1 and SUZ12 knockdowns, a sample for the non-targeting (shNT) line is shown for comparison to assess level of knockdown. (**B**) RT-QPCR showing mRNA levels for EBNA3C-activated or -repressed genes assessed for time points indicated. These were normalized to mRNA levels of endogenous control GNB2L1. The normalized values relative to *t* = 0 for each mRNA were plotted for cells without BMI1 knockdown (non-targeting (shNT)) and with BMI1 knockdown (shBMI1) after addition of HT and after HT wash, as indicated. Error bars represent standard deviation from three replicate QPCRs. (**C**) As in (B) but for shNT and shSUZ12.

The stable knockdowns of BMI1 and SUZ12 were used to directly assess the contribution of these proteins to EBNA3C-mediated regulation. HT was added to half the culture of LCL 3CHT with shBMI1, shSUZ12 or shNT. After 21 days, HT was washed from one half of each culture grown with HT and all cultures were grown for another 21 days, i.e. a total of 42 days. Cells were harvested and mRNA extracted for RT-QPCR at *t* = 0, 14, 21, 35 and 42 days. mRNA levels for each of the 12 EBNA3C-regulated genes chosen were assessed for each time point and normalized using levels for endogenous control GNB2L1, whose levels remained unaffected during the time course (Supplementary Figure S4B). Values from cells after addition and after washing of HT were plotted relative to *t* = 0 (Figure 7B). All six EBNA3C-activated genes were more activated relative to *t* = 0 in the non-targeting control, compared to cells with BMI1 knocked down (Figure 7B). Repression of EBNA3C-regulated genes in LCL 3CHT shBMI1 was either equal or better than in LCL 3CHT shNT (Figure 7B).

By the same analysis, when comparing LCL 3CHT shSUZ12 to LCL 3CHT shNT, SUZ12 knockdown did not appear to have an adverse effect on EBNA3C-mediated activation or repression (Figure 7C). In some cases, SUZ12 knockdown had an additive effect on EBNA3C regulation in cells with SUZ12 knocked down; there was more activation of AICDA, IL6R and ASCL1 and more repression of ZEB2 and, marginally, of PPM1L (Figure 7C), although it is possible this might be caused by the differences in steady state mRNA levels observed between shSUZ12 and shNT (Figure 6C). However, it is clear that EBNA3C can regulate its target genes at least as well in the shSUZ12 LCL as in the shNT LCL.

The consistent difference in the steady state mRNA levels between shBMI1 and shNT LCLs for EBNA3C-activated genes (Figure 6B) could theoretically make 3CHT appear less able to activate in shBMI1 compared to shNT (Figure 7B), because these genes are already activated by the BMI1 knockdown (Figure 6B) and might be unable to be activated further.

### BMI1 is required only for EBNA3C-mediated activation

**Figure 8.**
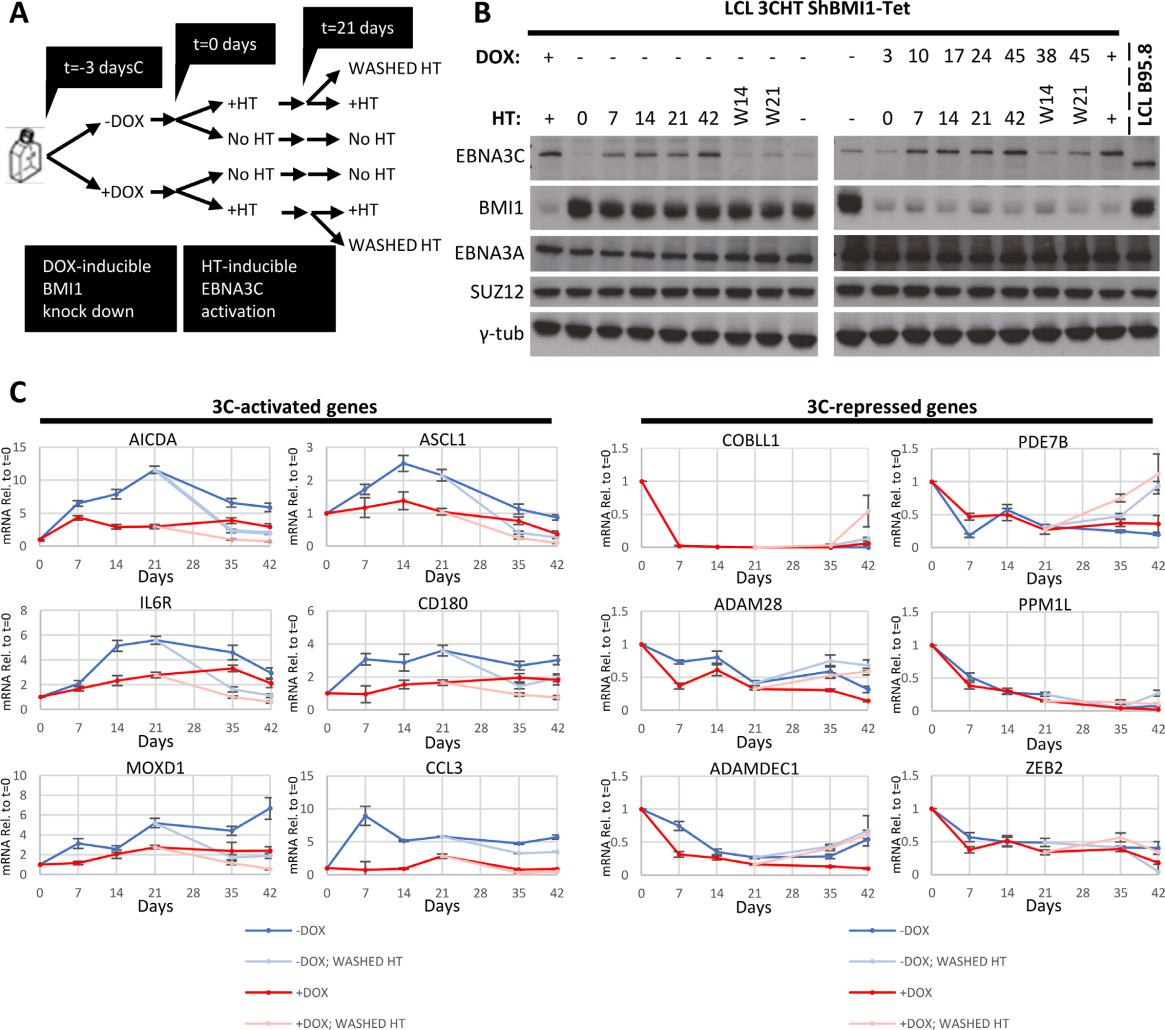
Effect of BMI1 inducible knockdown on host gene regulation by EBNA3C. LCL 3CHT was stably infected with lentivirus that constitutively expresses tetracycline repressor (TetR). ShRNA targeting BMI1 mRNA can be expressed from an H1 promoter upstream of two Tet Response Elements (TRE). In the absence of tetracycline analogue doxycycline (DOX), transcription is prevented allosterically by binding of TetR to the TREs. Addition of DOX changes the conformation of TetR, which can no longer bind TRE and transcription of BMI1 shRNA is possible in this inducible knockdown system (shBMI1-Tet). (**A**) Schematic of the time-course experiment: DOX was added to half the culture of LCL 3CHT shBMI1-Tet to induce expression of shRNA against BMI1, resulting in two cultures as shown. After 3 days, HT was added to half of each culture to activate 3CHT, resulting in four cultures as shown. Twenty-one days after addition of HT, HT was washed from half the cultures grown in its presence. Days are denoted relative to HT addotion. (**B**) EBNA3C protein activation and stabilization, as well as degradation in response to HT addition or washing was verified by western blots as was BMI1 knockdown after addition of DOX. SUZ12 and EBNA3A levels were also assessed through the time course and γ-tubulin was used as loading control. Days after DOX addition and after HT addition or washing are indicated seperately. (**C**) mRNA for EBNA3C-activated or -repressed genes was assessed for the time points indicated (relative to HT addition) from cells grown in the presence of HT and after washing of HT. These were normalized, first to mRNA levels of endogenous control GNB2L1 and then to similarly normalized values for the same genes from cells grown in the absence of HT throughout.

We wanted to assess EBNA3C-mediated regulation without the complicating effects of long-term stable knockdowns, such as already activated genes or selection of cells less amenable to regulation. To do this, a lentiviral system (pLKO-Tet-On) for doxycycline (DOX) inducible knockdown of BMI1 was used (45) to knock down BMI1 in LCL 3CHT (LCL 3CHT-Tet) and these cells were used in a time-course experiment (Figure 8A). The LCL 3CHT shBMI1-Tet culture was split in two and DOX was added to one half to knock down BMI1. Three days later, HT was added to half of each of these two cultures to activate 3CHT and 21 days after addition of HT, HT was washed out and cultures with all combinations of HT and DOX were followed for 21 days further (Figure 8A). Protein levels were tested by western blot for cells cultured with HT—and activated 3CHT—(Figure 8B) and for cells cultured without HT (Supplementary Figure S5A) to confirm the knockdown of BMI1 with addition of DOX, the activation and stabilization of 3CHT. EBNA3A and PRC2 core subunit SUZ12 levels remained unaltered throughout the time course (Figure 8B and Supplementary Figure S5A).

RT-QPCR was performed to quantify mRNA and measure expression of EBNA3-activated and -repressed genes at the time points indicated (Figure 8C). To assess 3CHT-mediated regulation at each time point in response to HT addition, values were first normalized with the endogenous control gene GNB2L1 and then with the GNB2L1-normalized values from cells cultured without HT, at the same time point (Figure 8C). In this way, at each time point the effects of the BMI1 knockdown that are not related to the action of 3CHT are being corrected and not allowed to influence the analysis. GNB2L1 expression did not change significantly during the time course.

For all EBNA3C-activated genes, knockdown of BMI1 abrogates the ability of 3CHT to activate, whereas for EBNA3C-repressed genes, 3CHT is able to repress in cells with BMI1 knocked down at least as well as in cells without BMI1 knockdown (Figure 8C). This indicates that BMI1 is important in EBNA3C-mediated activation, but not important for EBNA3C-mediated repression. This is the first factor shown functionally to influence host gene activation mediated by any EBNA3 protein.

## DISCUSSION

In this study, ChIP-seq was performed on an LCL for BMI1 and SUZ12, subunits of PRC1 and PRC2, respectively—two protein complexes shown to be critically important in the regulation of genes involved in differentiation, proliferation and cancer development. These new data sets can be used to compare the genomic localization of these important factors with the localization of other factors publicly available for ENCODE project Tier 1 cell line LCL GM12878 [https://www.encodeproject.org; (46)].

These ChIP-seq experiments were carried out in cells with the same genetic background as cells used in a comprehensive ChIP-seq study exploring the localization of EBV latent proteins EBNA3A, EBNA3B and EBNA3C (22). Past studies had suggested or questioned possible relationships between the EBNA3s and both PRC1 and PRC2, but many unanswered questions remained [reviewed in (7)]. A stepwise approach that started with simple co-localizations between BMI1 and SUZ12 with histone modifications and the EBNA3s led to the discovery of a functional relationship between EBNA3C and BMI1, identifying a general principle for EBNA3C-mediated activation that holds true for several loci.

Histone modifications and chromatin state (Figure 1) showed that BMI1 associates with active enhancers and promoters, something seen previously for EBNA3 proteins (21,22,55). In contrast, SUZ12 associated with chromatin of different characteristics (Figure 1), indicating less close association with the EBNA3s.

The difference between BMI1 and SUZ12 in distribution is compatible with current understanding of PRC1 and PRC2. It has been found previously that in differentiated cells the great majority of PRC1 complexes, including PCGF4/BMI1-containing PRC1, do not localize with H3K27me3 (56,57). In one study (57), using myelogenous leukaemia cell line K562 and normal fibroblasts Hs68, ChIP-seq data for RING1B and H3K27me3 were used to assess the differences in global distribution of PRC1 and PRC2 in differentiated cells. BMI1 data was only available for K562 cells, showing the same trend of difference between BMI1 and H3K27me3 localization. Here we show that in LCLs BMI1 does not colocalize with H3K27me3 and that BMI1 and SUZ12 are found at genomic loci with different chromatin characteristics (Figure 1), indicating that the trend extends beyond K562 cells.

In LCLs BMI1 extensively co-localizes with EBNA3 transcription factors (Figure 2). SUZ12 did not co-localize with any EBNA3 (Figure 2), in agreement with previous ChIP-seq studies that have shown EBNA3s do not localize with SUZ12-related H3K27me3 (21,22), but potentially at odds with many studies that have shown H3K27me3 accumulation around a number of EBNA3-repressed genes (8–10,12–15). H3K27me3 deposition was found to occur after establishment of repression around a handful of EBNA3A- and EBNA3C-regulated genes that have been tested in this way (9,10), suggesting this histone mark is a consequence of repression at these specific loci. Regardless of timings, the discrepancy between the presence of PRC2 or H3K27me3 around a few known EBNA3-regulated genes and the absence of PRC2/H3K27me3 at EBNA3-binding sites could have been due to EBNA3 binding at sites distal to regulated genes (21,22,58) and PRC2 possibly localized proximal to them, at their promoters. We found SUZ12 was present in ∼20% of the promoters of EBNA3A-repressed genes but absent from promoters of EBNA3C-repressed genes, as well as promoters of activated genes (Figure 3B). H3K27me3 was found before at EBNA3A-repressed genes (9) and an EBNA3A-repressed gene (STK39) where establishment of repression that is dependent on PRC2 has been described before (8). Therefore, suggestive evidence for the importance of PRC2 in the establishment of EBNA3A-mediated repression already exist from global data and the specific paradigm of STK39. For EBNA3C, such evidence is still lacking.

The same analysis for BMI1 revealed that it co-localizes more with EBNA3A and EBNA3C peaks than with the promoters of the genes these EBNA3s regulate (Figure 3A). Interestingly, significant co-localization was found for peaks associated with activated compared to repressed genes. The co-localizations observed also suggested that a physical interaction between BMI1 and EBNA3A and/or EBNA3C is possible.

Immunoprecipitations of BMI1 co-precipitated significant amounts of EBNA3C, verifying a physical interaction between these proteins (Figure 3). The minimal amount of EBNA3A co-precipitating with BMI1 (Figure 3) suggests that this might happen through EBNA3C, since EBNA3A and EBNA3C have been shown to physically interact (12). The indirect nature of the relationship between EBNA3A and BMI1, through EBNA3C, was further supported by ChIP experiments. BMI1 was recruited by EBNA3C at sites around all EBNA3C-regulated genes tested, but at only one of the EBNA3A-regulated genes, ALOX5AP, by EBNA3A (Figure 5). The fact that EBNA3C also regulates ALOX5AP, that EBNA3C is present at all these loci and that 3CHT activation leads to BMI1 recruitment to all loci (EBNA3C- and EBNA3A-regulated, Supplementary Figures S1 and S2 and Figure 5)—indicates that EBNA3C is the driver of recruitment of BMI1 at all these loci.

Stable knockdown of BMI1 or SUZ12 showed that these two factors exert an effect on EBNA3C-repressed loci independently of EBNA3C and in a locus-specific manner (Figure 6). Conversely, the same BMI1 knockdown caused a consistent effect on EBNA3C-activated genes, resulting in activation, in the absence of active EBNA3C, for all six genes tested (Figure 6B). These data indicated that BMI1 played a prominent and consistent role in regulating EBNA3C-activated genes and that the process was different to BMI1 just being recruited by EBNA3C.

Activation of 3CHT in LCL with SUZ12 knocked down showed conclusively for the first time that SUZ12, and by extension PRC2 (59,60), is not important in establishment of EBNA3C-mediated regulation (Figure 7C). The current model for PRC2 globally, independently of EBV, is that its recruitment is a consequence of repression establishment (61) and our data show that EBNA3C does not change this, even though EBNA3A might (8).

Surprisingly, 3CHT repressed equally efficiently with or without BMI1 knocked down in time courses, but 3CHT-mediated activation was consistently compromised with BMI1 knockdown (Figure 7B). Despite the differences in the starting points of gene expression levels between cells with or without BMI1 knocked down (Figure 6B: EBNA3C-repressed), there was remarkable similarity in the rate of repression for all EBNA3C-repressed genes, regardless of BMI1 status. Therefore, BMI1 is not necessary for the establishment of EBNA3C repression.

In addition, the results presented here suggest that the role of BMI1 (or SUZ12) in the maintenance of EBNA3C-mediated repression is limited and certainly not universal, because de-repression is not consistently more rapid or more significant in the knockdowns after inactivation of 3CHT in the later stages of the time courses (Figures 7B,C and 8C). However, in these time courses 3CHT was inactivated after only 21 days of active 3CHT being expressed in cells, which might not be enough for full establishment of repression. We cannot exclude the possibility that BMI1 or SUZ12 could become important in maintenance after longer periods of continuous repression.

For EBNA3C-activated genes, the consistent impairment of activation with BMI1 knockdown shown in Figure 7 is striking but could be an artefact due to the consistent difference in the starting points of gene expression between cells with or without the knockdown. BMI1 knockdown leads to increased expression of all these genes in cells grown without HT (Figure 6B) and 3CHT-mediated activation could seem impaired because gene expression has reached the maximum possible for these loci before the start of the time course. This possibility was addressed by employing a conditional BMI1 knockdown that allowed testing 3CHT-mediated regulation immediately after reduction in BMI1 levels, before activation could reach a maximum. This also minimized the effects of indirect regulation at the chosen loci. Under these conditions, EBNA3C-mediated activation was again found to be compromised after BMI1 knockdown (Figure 8C). This is the first direct demonstration of a factor being involved in EBNA3-mediated activation of genomic loci and shows that activation by EBNA3C is achieved, at least in part, through BMI1. EBNA3C was again able to repress all six genes tested after BMI1 knockdown at least as well as in the non-targeting control (Figure 8C), confirming that BMI1 is not important for repression.

BMI1 is a core component of PRC1, which is mainly associated with repression (62). Previous work had shown that knockdown of BMI1 in LCL resulted in some de-repression of EBNA3C- (and EBNA3A-) repressed *BIM* (12). Moreover, BMI1 accumulation was observed around EBNA3C-repressed genes with addition of EBNA3C (10), something that was observed again here for all loci of EBNA3C occupancy tested (Figure 5). Based on all this, BMI1 was considered as being important for EBNA3C-mediated repression. The experiments presented here show that this interpretation, although reasonable at the time, is incorrect. BMI1 recruitment by 3CHT appears to be misleading because activation of genes tested could be achieved either by knockdown of BMI1 in the absence of active 3CHT or in the presence of active 3CHT and recruitment of BMI1 at the same loci. Higher expression of EBNA3C-activated genes after BMI1 knockdown in the absence of 3CHT (Figure 6B) and impaired ability of 3CHT to activate following BMI1 knockdown (Figure 8C) suggest that it is the presence of repressive BMI1 keeping expression of these genes low and that 3CHT somehow counters the presence of repressive BMI1 to mediate repression.

Gene activation by EBNA3C is likely to involve PRC1’s ability to monoubiquitinate H2AK119. Monoubiquitinated H2AK119 could not be immunoprecipitated from LCL chromatin by ChIP using four different antibodies, despite being detected by western blot, which could reflect a particular characteristic of LCLs relative to this histone modification. There have been some studies that describe PRC1 complexes mechanistically involved in (63–66) or associated with activation (67,68). Of these studies, the one by Frangini *et al.* (65) could be the most relevant to data presented here. They showed that a PRC1 complex variant that contains BMI1 is present at active genes in quiescent B cells isolated from mouse spleen. They also showed that Aurora B kinase is responsible for activation in this context because it prevents H2AK119 ubiquitination, which is important for repression by PRC1 (29). It is known that EBNA3C can be found in complex with Aurora B (69) and this might mean that EBNA3C causes gene activation by preventing PRC1-mediated H2AK119 ubiquitination through its association with Aurora B.

Therefore, in seeking to assess the importance of BMI1 and SUZ12 as proxies for canonical PRC1 and PRC2 in gene regulation by the EBNA3s, we produced data that suggest that EBNA3A and EBNA3B are not associated with either, but EBNA3C is the main interactor with BMI1. Since EBNA3C can repress effectively with BMI1 or SUZ12 knocked down, current available evidence suggest that histone deacetylation might be the driver for repression (this study and evidence summarized in (7)). Data presented here show for the first time that EBNA3C activates genes by restricting the action of a repressive BMI1.

## DATA AVAILABILITY

The ChIP-seq data reported in this paper have been deposited in the GEO database under accession number GSE119823.

## Supplementary Material

Supplementary Data
