# Expression of Concern on ‘Requirement for PRC1 subunit BMI1 in host gene activation by Epstein–Barr virus protein EBNA3C’

**DOI:** 10.1093/nar/gkag940

**Published:** 2026-09-28

This is an Expression of Concern regarding: Kostas Paschos, Quentin Bazot, Jonathan Lees, Paul J Farrell, Martin J Allday, Requirement for PRC1 subunit BMI1 in host gene activation by Epstein–Barr virus protein EBNA3C, *Nucleic Acids Research*, Volume 47, Issue 6, 08 April 2019, Pages 2807–2821, https://doi.org/10.1093/nar/gky1323

In March 2026, the Editors were made aware of concerns regarding potential image irregularities in Figures 4 and 6A of the above article. These concerns were also raised on PubPeer (https://pubpeer.com/publications/B300087240F78AC6E1639E5B4B0C32).

These concerns relate to the apparent duplication of immunoblot bands within the article and with a figure previously published in a different *Nucleic Acids Research* article, under different experimental conditions and depicting a different protein.

The Editors have contacted the authors and are escalating to the institution for investigation in line with COPE guidance. In the interim, the Editors are publishing this Expression of Concern to alert readers while the outcome of the investigation is pending and advise readers to examine the details of this study with particular care.

