# Supplementary material for: Requirement for PRC1 subunit BMI1 in host gene activation by Epstein–Barr virus protein EBNA3C
Source: Nucleic Acids Res. 2019 Jan 15;47(6):2807–21. doi: 10.1093/nar/gky1323 (PMC6451101; doi:10.1093/nar/gky1323)
Supplement: Supplementary Data [file gky1323_supplemental_files.zip › Suppl Figures_table_legends .pdf]

Supplementary Figure S1

A

ALOX5AP (EBNA3A-activated)

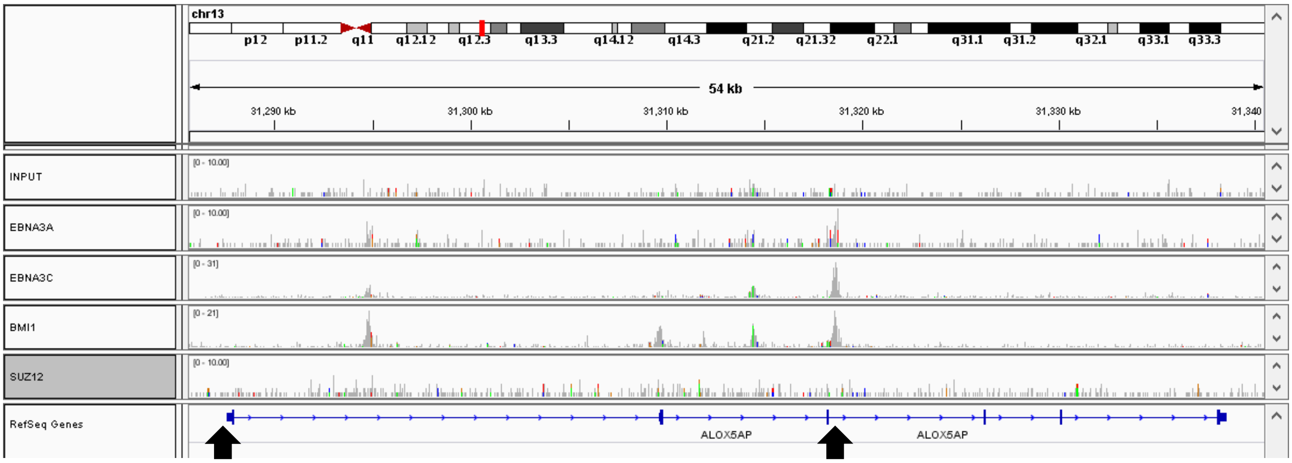

S100A10 (EBNA3A-repressed)

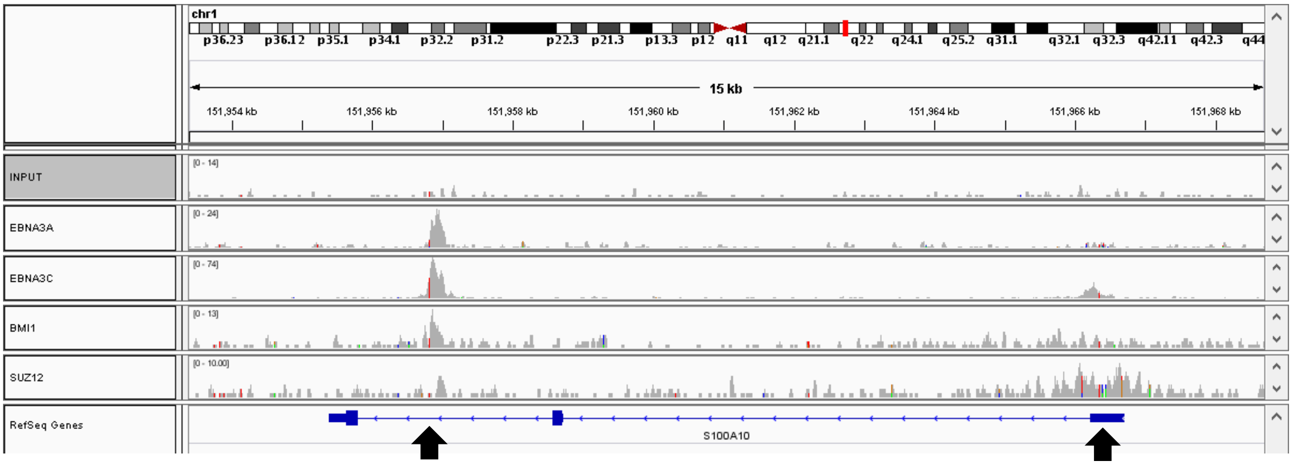

STK39 (EBNA3A-repressed)

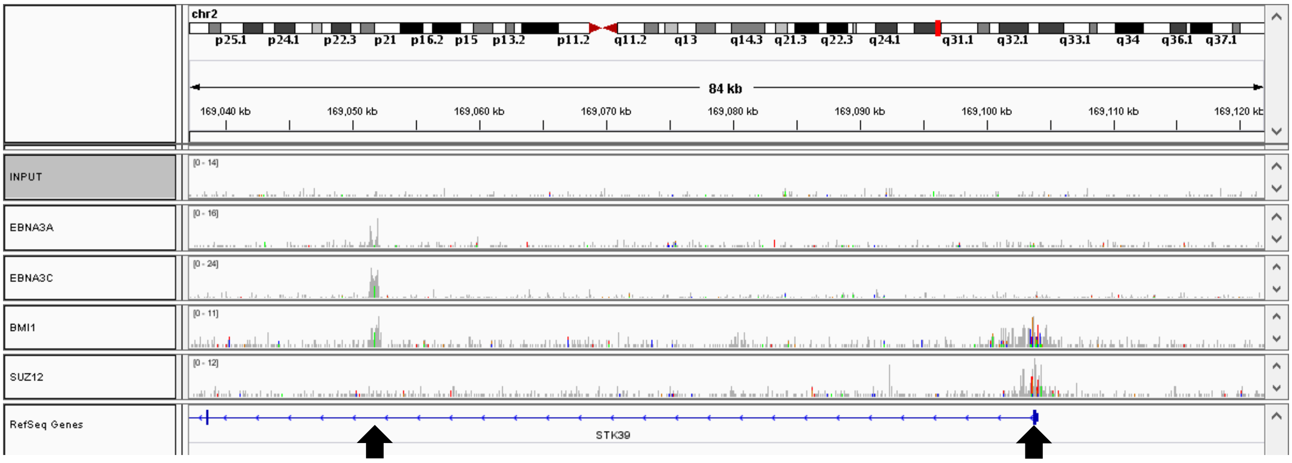

Supplementary Figure S1 (continued)

B

AICDA (EBNA3C-activated)

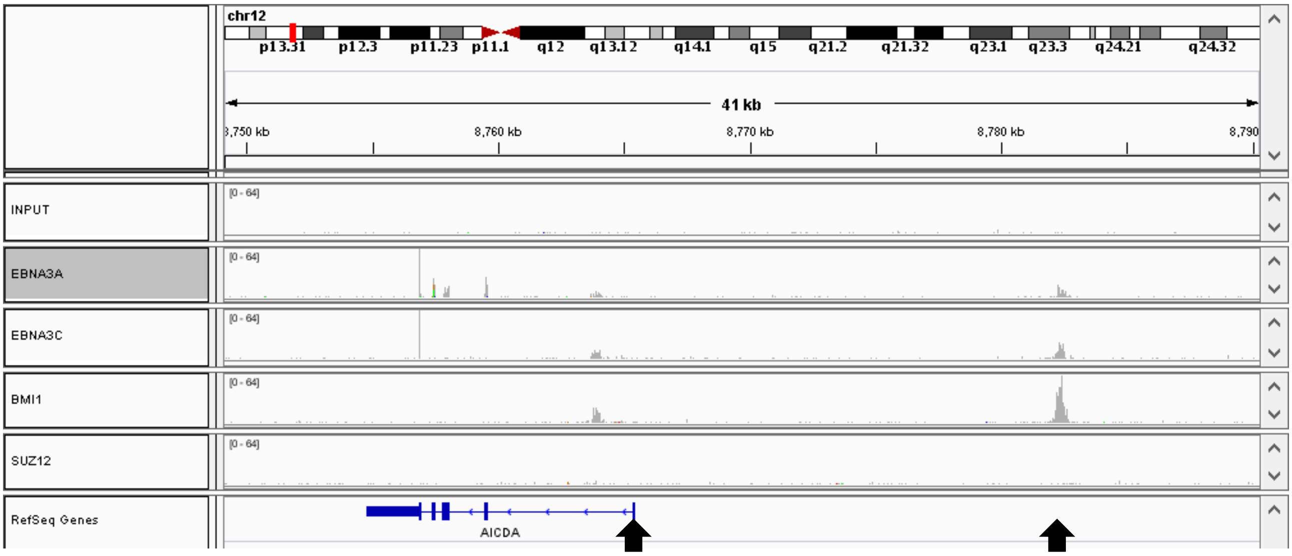

IL6R (EBNA3C-activated)

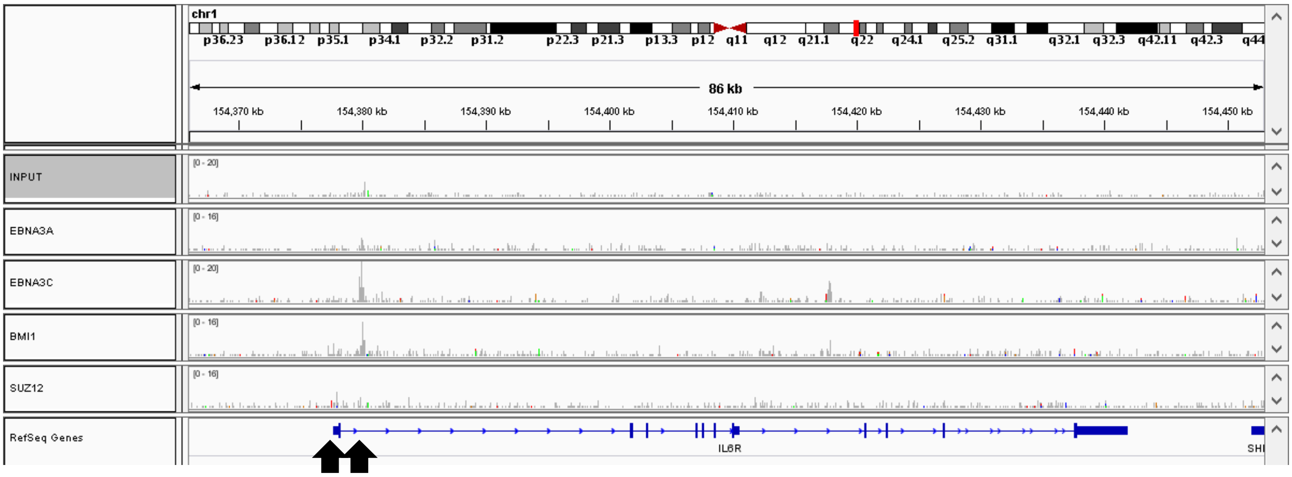

COBLL1 (EBNA3C-repressed)

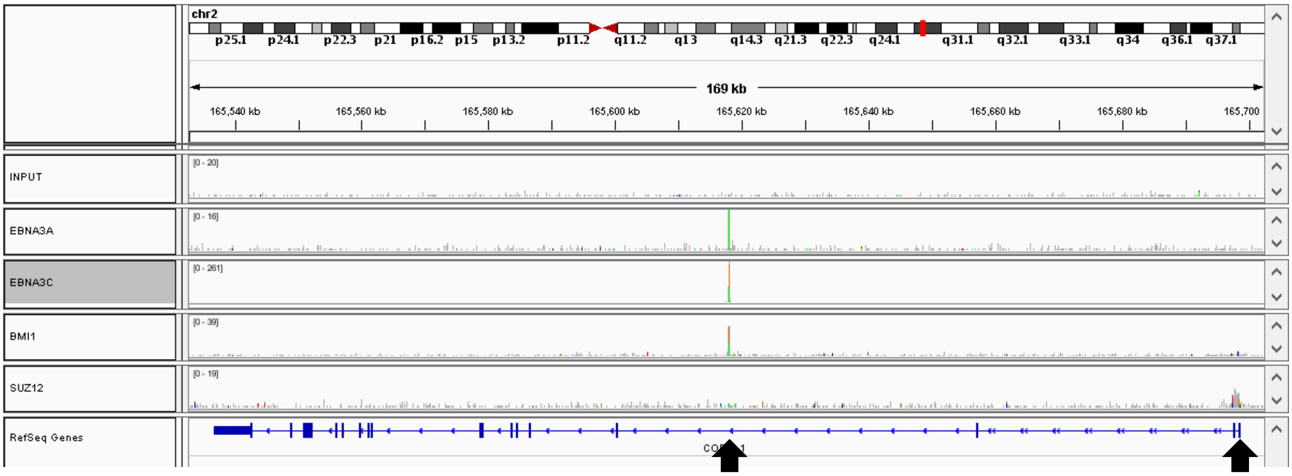

Supplementary Figure S2

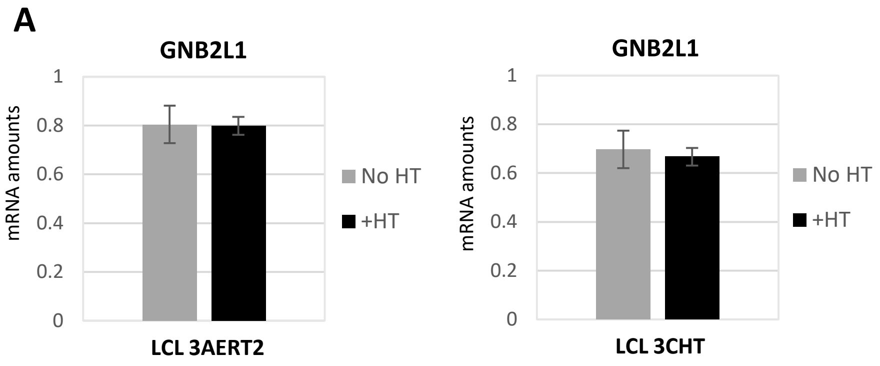

Supplementary Figure S2 (continued)

B

HPSE2

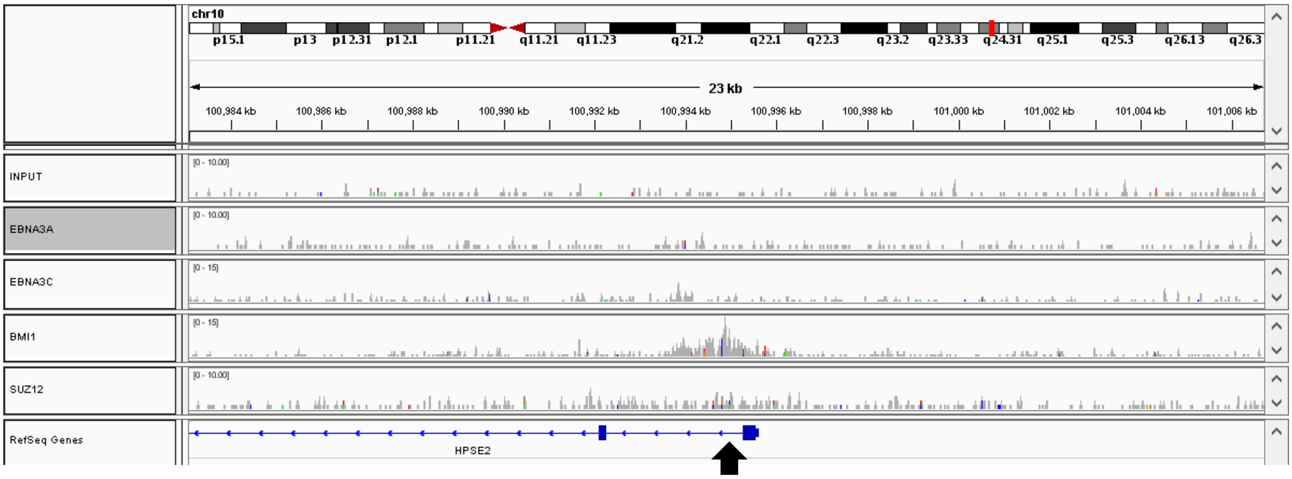

MCM6

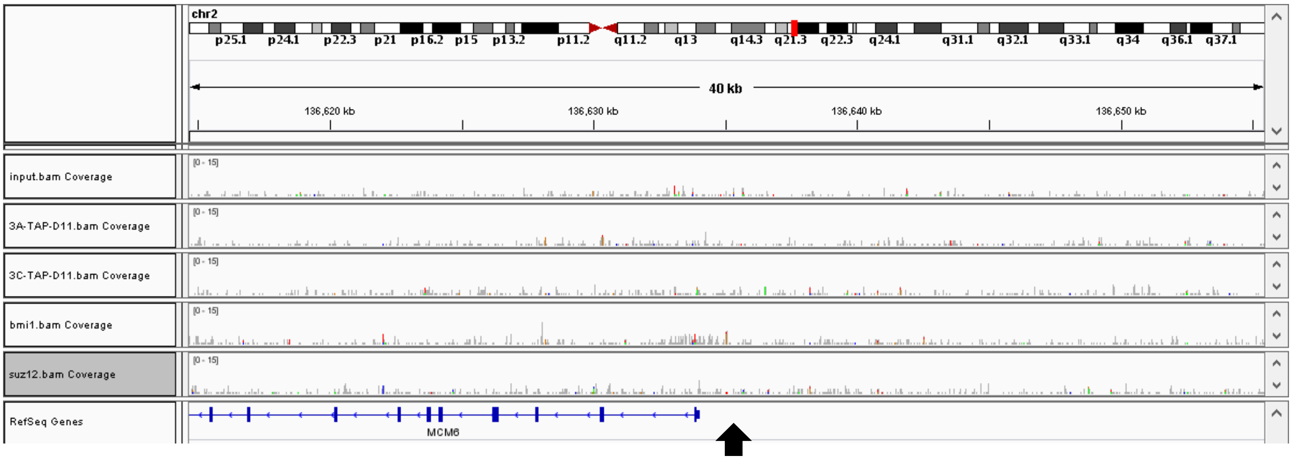

C

ALOX5AP

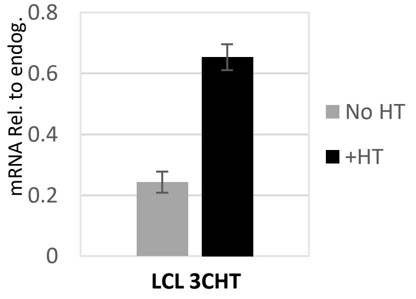

Supplementary Figure S3

MOXD1 (EBNA3C-activated)

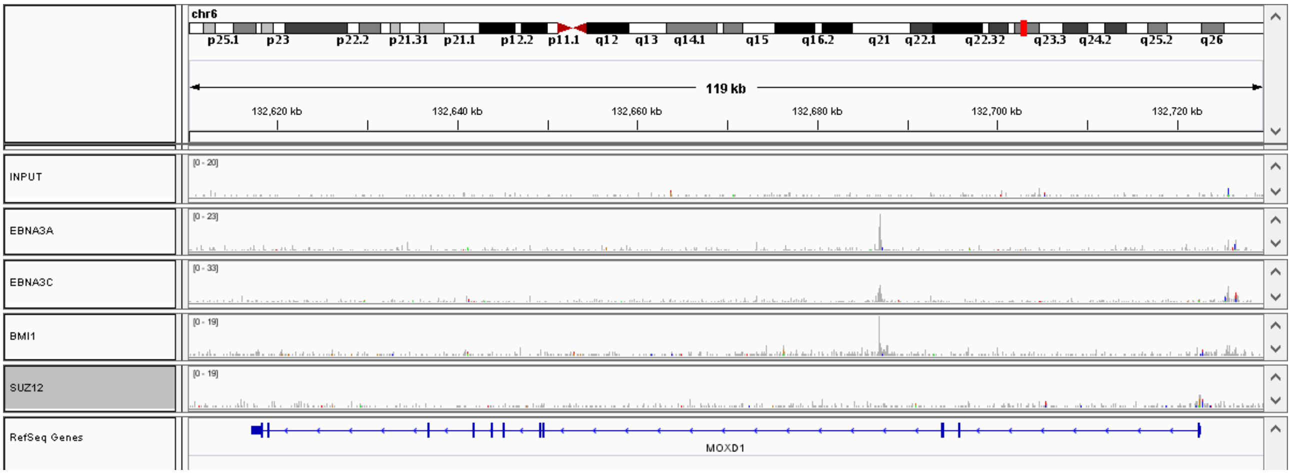

ASCL1 (EBNA3C-activated)

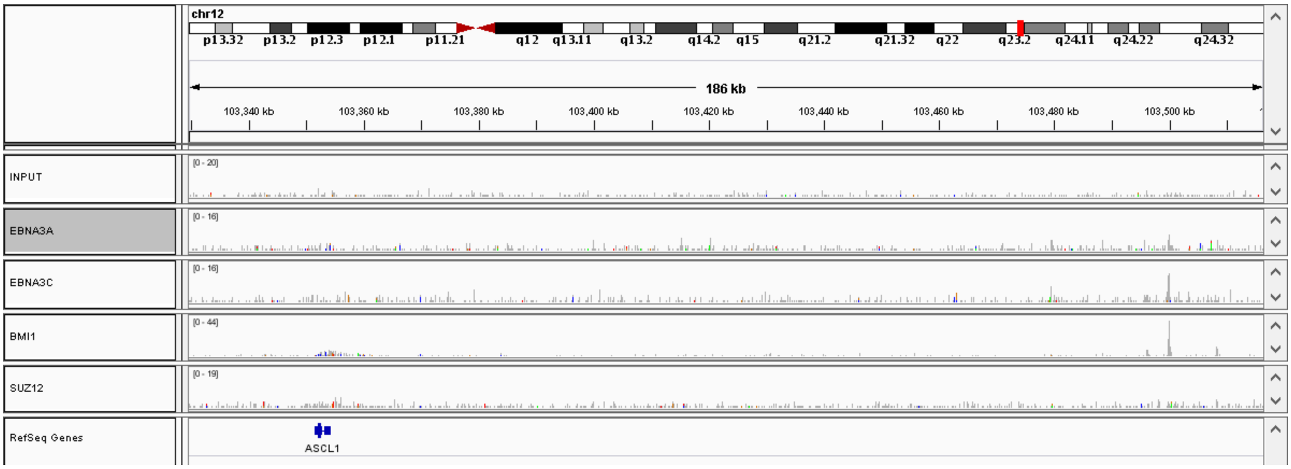

CD180 (EBNA3C-activated)

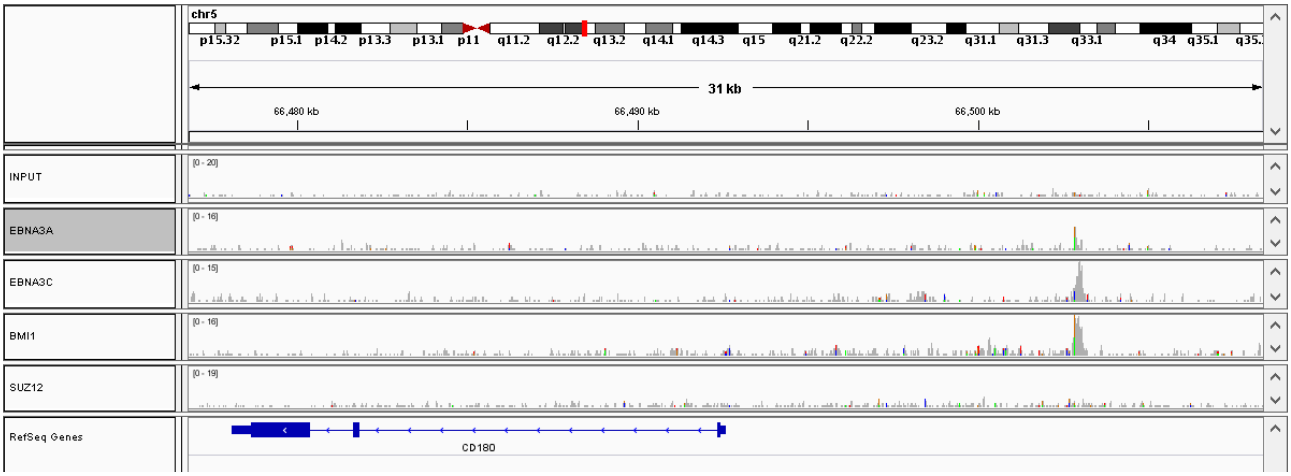

Supplementary Figure S3 (continued)

CCL3 (EBNA3C-activated)

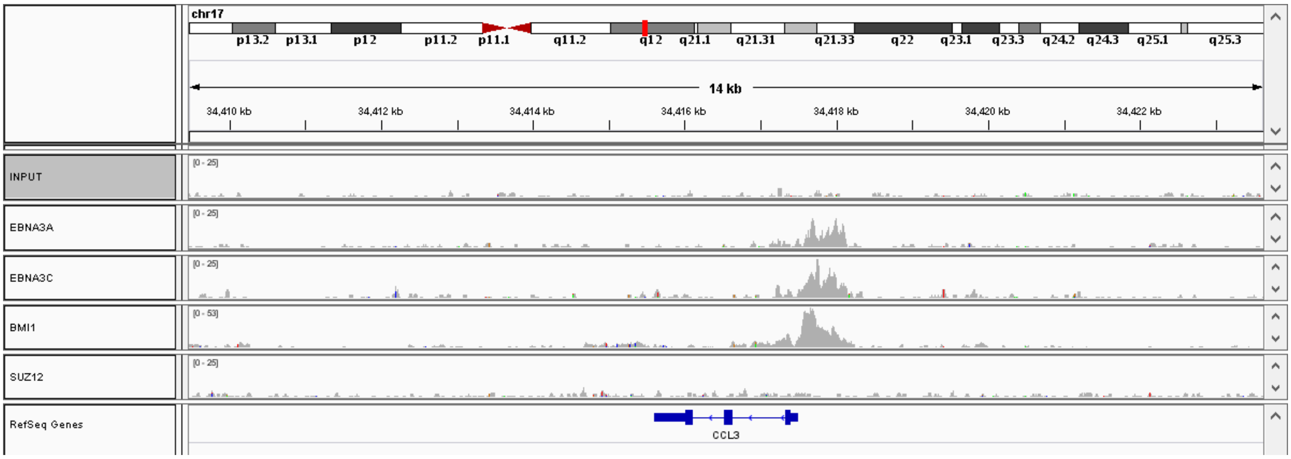

ADAM28-ADAMDEC1 (EBNA3C-repressed)

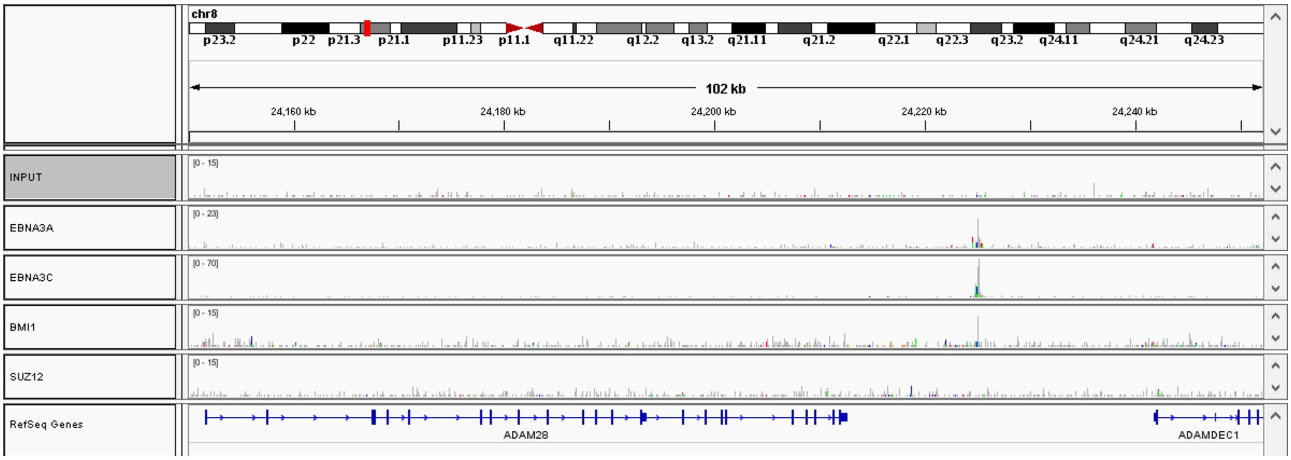

PDE7B (EBNA3C-repressed)

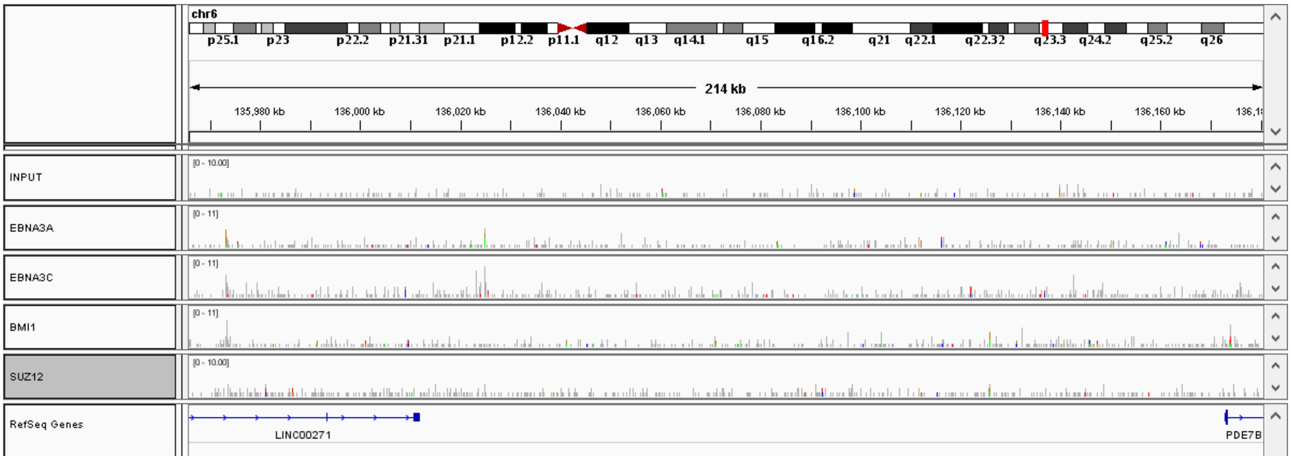

Supplementary Figure S3 (continued)

PPM1L (EBNA3C-repressed)

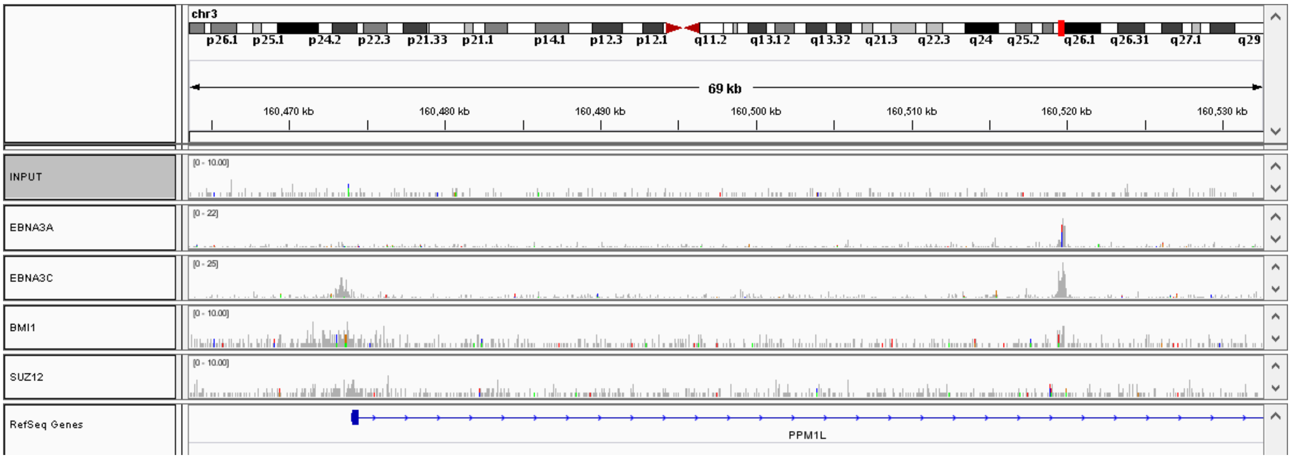

ZEB2 (EBNA3C-repressed)

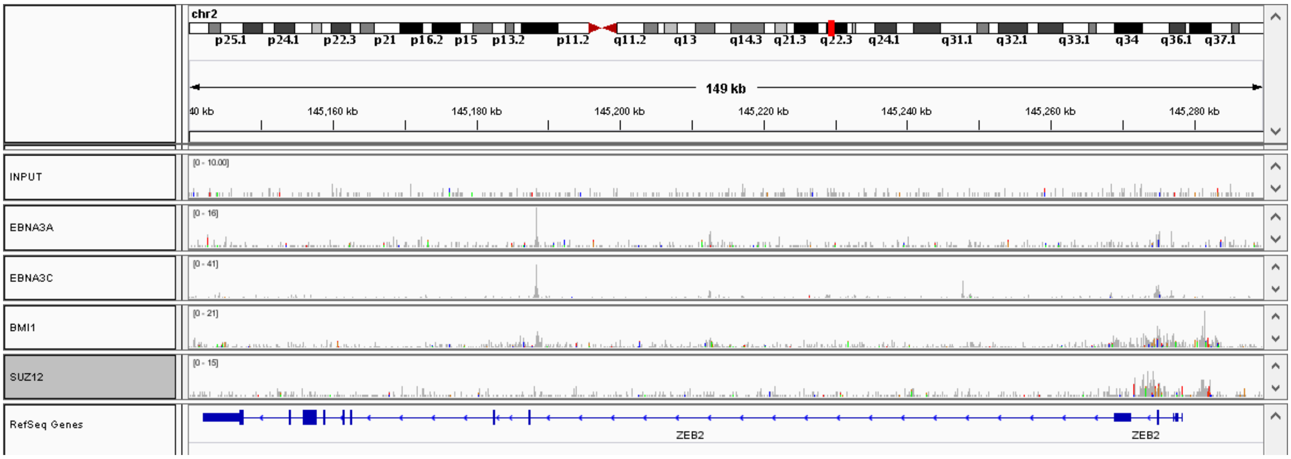

Supplementary Figure S4

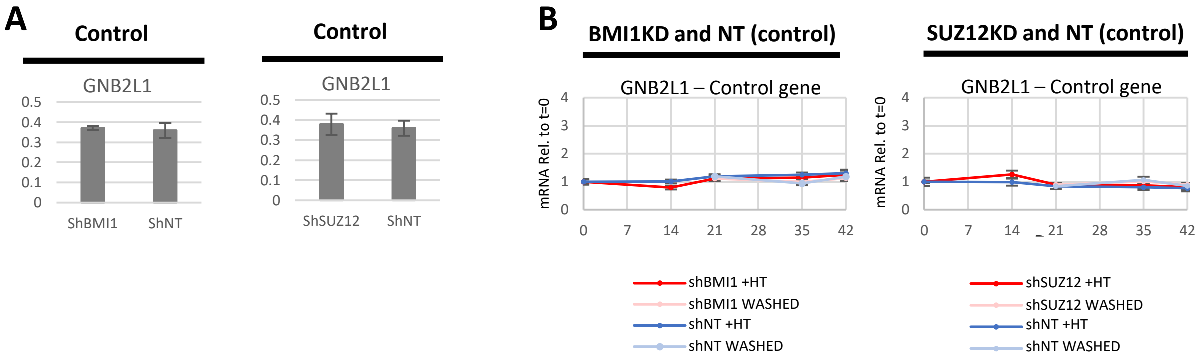

Supplementary Figure S5

A

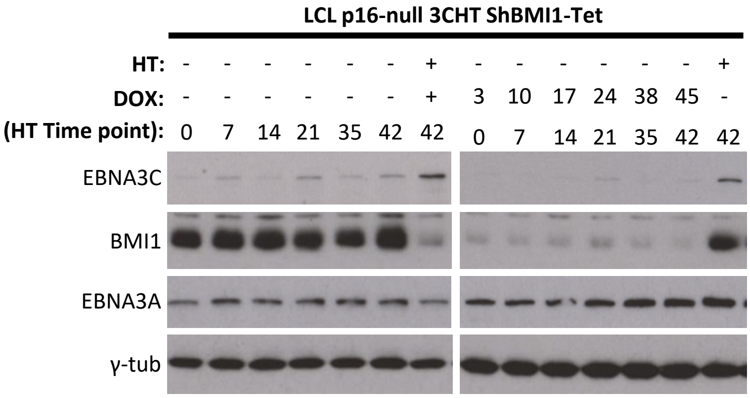

B

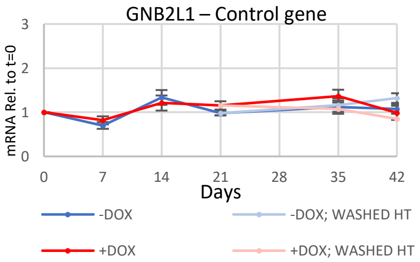

Supplementary Table S1

**Primers for quantifying cDNA by Q-PCR**		
**Gene**	**FW**	**REV**
ALOX5AP	TAAAGTGGAGCACGAAAGCA	CACAGTTCTGGTTGGCAGTG
S100A10	TGCCATCTCAAATGGAACAC	GCCCACTTTGCCATCTCTAC
STK39	CTCTGTGCACGACTCTCAGG	GAGCAAACCCAATCAGCTTC
AICDA	AGCCGTTCTTATTGCGAAGA	TGATGAACCGGAGGAAGTTT
IL6R	TTGTTTGTGAGTGGGGTCCT	TGGGACTCCTGGGAATACTG
COBLL1	CTGTTCAGCTGACAACAGATCG	ACGTTGAACTCTCAGTGGTCCT
MOXD1	GAACCTATCCGCACCGGACC	AGCCGAAGCCCACGTAGC
ASCL1	TGGTGCGAATGGACTTTGGA	CGCCACTGACAAGAAAGCAC
CD180	GCTTCTTTTGGGTGGTGCTG	TGTGTTTGGTAGAGTGTCAGGG
CCL3	TTCCGTCACCTGCTCAGAATC	GTGATGCAGAGAACTGGTTGC
ADAM28	GTTGCAGGGACAATGGCACA	TGAGACGGCTGCAGGAACTG
ADAMDEC1	CCTTGGTATGCCTGATGTTCCA	CAGCAGGCACTTTGGTTTCTGA
PDE7B	CTTCATGAACAAGCAGCACCG	GGCATTCTGATCGGGGTTCT
PPM1L	GCTGGACTTCACGATGGTCT	CTTTGCTGTCTCTGCTGGGT
ZEB2	CCCTGGCACAACAACGAGAT	GGTCTGGATCGTGGCTTCTG
ALAS1	TCCACTGCAGCAGTACACTACCA	ACGGAAGCTGTGTGCCATCT
GNB2L1	GCTTGCAGTTAGCCAGGTTC	GAGTGTGGCCTTCTCCTCTG
**Primers for quantifying DNA from ChIP**		
**Locus**	**FW**	**REV**
ALOX5AP Pk	TTCCTGTGGGGTTAGACCAG	GTGGGTGAGTTGAAGGCTGT
ALOX5AP TSS	GTCTGGGGTGTGTTTGGTCT	ACCTCCAAATGCCCAGATAA
S100A10 Pk	ACGCAGTGTTTCAAGTGTGC	ACCCACCAAGAGCTGCATAG
S100A10 TSS	TGCGCCTTCCTTAGTACGTG	AGGAAGAGGGGGAGGAGACA
STK39 Pk	TCACTTTTACATCCTCGTTGTCAAG	TTTGTTGCCCAGGTTATGTC
STK39 TSS	CAGCACCAACGCTCATAGAA	GGCCTACTGGCTTCAACAGT
AICDA Pk	TGGACACCAGCTAGATTGTTCA	TCACACTTTCACCCACACAGA
AICDA TSS	GAGGAAGGCCAGTGCAATCA	CAGGGAGGCAAGAAGACACT
IL6R Pk	GCTCCTGAGTGGATGCTAGG	CCCCAGTCTCTCAGTTCTGC
IL6R TSS	GTGTGCCTTTCACAGCGTAA	CTCCCGGTTCTGTTGATGAG
COBLL1 Pk	CTGAGTAACAAGAGCGAAAGAG	ATCAGATGTGTTATGACTAACAGC
COBLL1 TSS	GCCGCCGTCTCTACAAGGTCTA	CTACCCAGTAAACCCCACGG
HPSE2 TSS	TCGTTTTCTGAGAGTGGGAGA	GTGAGTGTTCAAGCGGGATT
MCM6 TSS	CTCTTTGGCCATTCCTGATT	GCCCAAGAGAGCGACTTTAC

Supplementary Figure S1. Aligned reads visualized using Integrated Genomics Viewer (IGV) from BMI1 and SUZ12 ChIP-seq presented in this study and EBNA3A and EBNA3C presented in a previous study (22). Reads from an input sample for the BMI1 and SUZ12 ChIP-seq are shown for comparison. Loci indicated by arrows were interrogated by ChIP-QPCR to assess BMI1 recruitment in response to EBNA3A (3AERT2) or EBNA3C (3CHT) activation. (A) Loci around genes identified as EBNA3A-regulated. (B) Loci identified as EBNA3C-regulated. These genes were also among 12 EBNA3C-regulated genes whose EBNA3C-mediated regulation was studied in time courses in response to EBNA3C activation with or without BMI1 or SUZ12 knock down.

Supplementary Figure S2. (A) mRNA levels of endogenous control gene GNB2L1, used for normalization, from cells grown in the absence of HT (No HT) and after 14 days cultured with HT (+HT) for LCL 3AERT2 and LCL3CHT as indicated. Height of bars represent not normalized amounts, extrapolated from a standard curve of a mix of mRNAs from the same experiment. Error bars are standard deviation from 3 replicate QPCR reactions. (B) Aligned reads visualized using IGV as in Supplementary Figure S1 for loci used as controls – positive and negative – for BMI1 ChIP-QPCR. Regions amplified are indicated by arrows. (C) mRNA levels of ALOX5AP – identified as an EBNA3A-activated gene – in LCL 3CHT. Height of the bars represents mRNA amounts normalized with amounts of endogenous control GNB2L1. Error bars are standard deviation from 3 replicate QPCR reactions.

Supplementary Figure 3. Aligned reads visualized using IGV as in Supplementary Figure S1 for loci around genes identified as EBNA3C-regulated and whose regulation in response to EBNA3C activation was followed in time courses with or without BMI1 or SUZ12 knock down.

Supplementary Figure S4. (A) RT-QPCR showing mRNA levels of endogenous control gene GNB2L1, used for normalization, from cells grown in the absence of HT (42 days time point) that were stably infected with lentiviruses expressing BMI1 shRNA (shBMI1), SUZ12 shRNA (shSUZ12) or non-targeting shRNA (shNT) as indicated. Height of bars represent not normalized amounts, extrapolated from a standard curve of a mix of mRNAs from the same experiment. Error bars are standard deviation from 3 replicate QPCRs. (B) RT-QPCR showing mRNA levels for endogenous control GNB2L1 assessed for the time points indicated. All values shown relative to levels at t=0 for each condition indicated. Error bars are standard deviation from 3 replicate QPCRs.

Supplementary Figure 5. (A) Western blots following protein levels in cells cultured in the absence of HT, at each time point as indicated. In each panel, protein from cells cultured in the presence of HT and with alternate DOX environment to the other samples in the same panel is shown (from the 42 days time point) for comparison between degraded or stabilised 3CHT and between expression or

not of the BMI1 shRNA. Γ-tubulin was used as loading control. (B) mRNA levels for endogenous control gene GNB2L1 were assessed by RT-QPCR for the time points indicated. All values shown relative to levels at t=0 for each condition indicated. Error bars represent standard deviation from 3 replicate QPCRs.

Supplementary Table S1. List of oligonucleotide primers used for QPRC
